# Epithelial CD47 is critical for mucosal repair in the murine intestine in vivo

**DOI:** 10.1038/s41467-019-12968-y

**Published:** 2019-11-01

**Authors:** Michelle Reed, Anny-Claude Luissint, Veronica Azcutia, Shuling Fan, Monique N. O’Leary, Miguel Quiros, Jennifer Brazil, Asma Nusrat, Charles A. Parkos

**Affiliations:** Department of Pathology, University of Michigan, Ann Arbor, MI 48109 USA

**Keywords:** Focal adhesion, Mucosal immunology, Inflammatory bowel disease, Gastrointestinal models

## Abstract

CD47 is a ubiquitously expressed transmembrane glycoprotein that regulates inflammatory responses and tissue repair. Here, we show that normal mice treated with anti-CD47 antibodies, and *Cd47*-null mice have impaired intestinal mucosal wound healing. Furthermore, intestinal epithelial cell (IEC)-specific loss of CD47 does not induce spontaneous immune-mediated intestinal barrier disruption but results in defective mucosal repair after biopsy-induced colonic wounding or Dextran Sulfate Sodium (DSS)-induced mucosal damage. In vitro analyses using primary cultures of CD47-deficient murine colonic IEC or human colonoid-derived IEC treated with CD47-blocking antibodies demonstrate impaired epithelial cell migration in wound healing assays. Defective wound repair after CD47 loss is linked to decreased epithelial β1 integrin and focal adhesion signaling, as well as reduced thrombospondin-1 and TGF-β1. These results demonstrate a critical role for IEC-expressed CD47 in regulating mucosal repair and raise important considerations for possible alterations in wound healing secondary to therapeutic targeting of CD47.

## Abstract (web summary)

The role of the transmembrane glycoprotein CD47 in healing injured intestinal mucosa is unclear. Here, the authors show that selective loss of CD47 in the murine intestinal epithelium results in defective mucosal repair after colonic wounding, with suggested impaired cell migration in vitro.

## Introduction

CD47 is a ubiquitously expressed cell surface glycoprotein that associates with a variety of receptors to facilitate critical cell signaling events. Of considerable interest, CD47 has been implicated to regulate innate immune cell tolerance of self through its ability to ligate signal-regulatory protein alpha/SIRPα on myeloid immune cells and inhibit phagocytosis of circulating CD47-expressing erythrocytes^1–6^. This mechanism is currently being exploited in studies on CD47 as a potential target for immunotherapeutics intending to augment immune-mediated destruction of cancer cells that overexpress CD47^7,8^.The altered susceptibility of *Cd47*-deficient mice to inflammatory insults further suggest that CD47 expression is an important modulator of leukocyte function during pro-inflammatory immune responses^9–14^. However, a lack of tissue-targeted knockout mice has limited studies that address the function of CD47 expression by non-hematopoietic cell types in vivo.

It is well appreciated that adhesion and collective migration of epithelial cells is particularly important during mucosal wound closure, as restoration of the epithelial barrier is necessary for the resolution of inflammation^15^. Studies suggest that collective migration of epithelial cell monolayers across denuded surfaces requires dynamic regulation of β1 integrin signaling, which directs the rapid formation and dissolution of focal adhesions^16–18^. In vitro studies using cell lines suggest that CD47 is also an important regulator of integrin signaling through in cis interactions with integrin heterodimers. Specifically, it has been reported that CD47 expression regulates integrin-dependent cell adhesion and migration in smooth muscle cells, platelets, and epithelial cells in vitro^19–22^, as well as enabling integrin-mediated leukocyte adhesion and extravasation in vivo^23^. However, the potential regulation of epithelial β1 integrin signaling by CD47 during mucosal wound closure in vivo has not been investigated. Noteworthy, CD47 has been reported to play a role in tissue repair with improved healing and survival in several in vivo models including skin thermal injury and ischemia/reperfusion in organ transplant models of liver, kidney, and skin^24–29^. Given these observations, we surmised that expression of CD47 in epithelial cells may play an important role in regulating mucosal wound closure in vivo. Studies were performed to evaluate the role of epithelial CD47 expression in models of intestinal mucosal injury and repair in vivo. Here, we report that *Cd47*-deficient mice and wild-type animals infused with CD47-neutralizing antibodies have markedly impaired biopsy-induced colonic wound closure. We further demonstrate a critical role of epithelial expressed CD47, as mice selectively deficient in intestinal epithelial cell (IEC) CD47 expression did not display abnormalities under resting conditions but exhibited profound defects in closure of biopsy-induced mucosal wounds as well as markedly impaired colonic mucosal wound repair from cyclic exposure to dextran sodium sulfate (DSS)-induced injury. Using two-dimensional (2D) cultures of primary epithelial cells derived from murine and human intestine, it was determined that CD47 regulates epithelial cell migration and wound closure through β1 integrin-dependent signaling through Focal adhesion kinase (FAK), Src protein-tyrosine kinase, and Crk-associated substrate p130Cas (p130Cas), thereby controlling the formation of focal cell matrix adhesions. In addition, we observed that loss of CD47 negatively regulates the level of expression of its ligand thrombospondin-1 (TSP-1) and effector cytokine Transforming growth factor beta (TGF-β1) both known to facilitate wound healing^30–32^, resulting in alteration of downstream signaling pathways. Herein, we corroborate evidence of crosstalk between CD47, β1 integrin, and focal adhesions as well as a functional link between CD47, TSP-1, and TGF-β1 in the intestinal epithelium. These findings provide insights into a central role of CD47 in regulating IEC migration and mucosal wound repair in vivo, and raise the possibility of altered wound healing as a potential complication in clinical studies exploring blockade of CD47 to enhance clearance of CD47-overexpressing tumor cells in humans.

## Results

### CD47 expression is required for mucosal repair in vivo

**Fig. 1:**
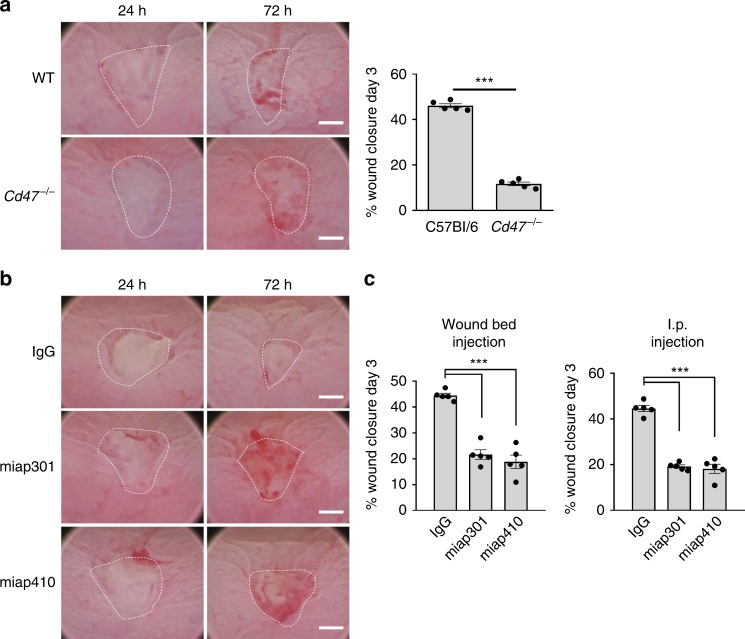
CD47 regulates mucosal wound healing in vivo. Utilizing a miniature video endoscope and biopsy scissors, 5–7 wounds were created in the dorsal aspect of the descending colon mucosa of anesthetized mice. **a** Digital measurement of wound surface area at 24 and 72 h post wounding revealed a striking impairment in wound closure in *Cd47*^−/−^ mice. Points represent the mean value within all wounds from individual mice. Data are representative of three independent experiments with five mice per group and are expressed as means ± SEM. ***p < 0.001; two-tailed Student’s *t* test. **b** In total, 10 µg of control antibody (IgG) or anti-CD47 antibody (miap301 or miap410) were injected into wound beds of wounds created 24 h previously in C57Bl/6 mice, resulting in substantial reduction of wound closure upon blockade of CD47. **c** Mice treated locally or systemically with anti-CD47 monoclonal antibodies miap301 or miap410 experienced less wound area reduction in comparison with IgG-treated controls. Points represent mean value within all wounds from an individual mouse. Data are representative of two independent experiments with five mice per group. Date are means ± SEM. ***p < 0.001; one-way ANOVA. Scale bars: 50 mm. Source data are provided as a Source Data file

Previous studies with total *Cd47* knockout mice (*Cd47*^−/−^) have shown varied results in response to inflammatory challenge, including a protective effect of CD47 deficiency during ischemia/reperfusion injury and total body irradiation^13,28^, as well as in response to certain pathogens^33^. However, CD47 deficiency has also been reported to increase susceptibility to systemic *Escherichia coli* and *Candida albicans* infection^10,11^. As mucosal wound repair in the intestine requires coordinated epithelial migration and proliferation in concert with temporal immune responses^15^, we hypothesized that CD47 may have an important role in regulation of wound healing. To test this hypothesis, we utilized a mucosal repair model employing miniaturized endoscopic biopsy-based mechanical injury and video analysis of recovery in the colonic mucosa of *Cd47*^−/−^ mice. To analyze repair, sizes of mucosal wounds were digitally quantified 1 day after initial wounding and compared with sizes of the same wounds 2 days later. *Cd47*^−/−^ mice showed markedly inhibited wound closure compared with wild-type controls (Fig. 1a).

Although the above results suggest that loss of CD47 impairs mucosal wound closure, recent studies employing systemic blockade of CD47 with monoclonal antibodies suggest beneficial effects in some murine models of inflammation^34,35^. We therefore tested whether systemic or local administration of CD47-blocking antibodies had inhibitory effects on mucosal wound healing. Indeed, intraperitoneal injection of the CD47-blocking antibodies miap301 or miap410 significantly inhibited wound closure in C57Bl/6 mice (Fig. 1c). As systemic blockade of CD47 would be expected to alter immune cell extravasation^23^, we locally injected CD47-blocking antibodies directly into colonic wound beds 24 h post wounding and assessed wound closure. Similar results to those observed with systemic antibody administration as well as in *Cd47* knockout mice were obtained (Fig. 1b, c), suggesting that both localized and systemic neutralization of CD47 significantly delay mucosal wound healing.

### Loss of epithelial CD47 impairs wound healing responses

**Fig. 2:**
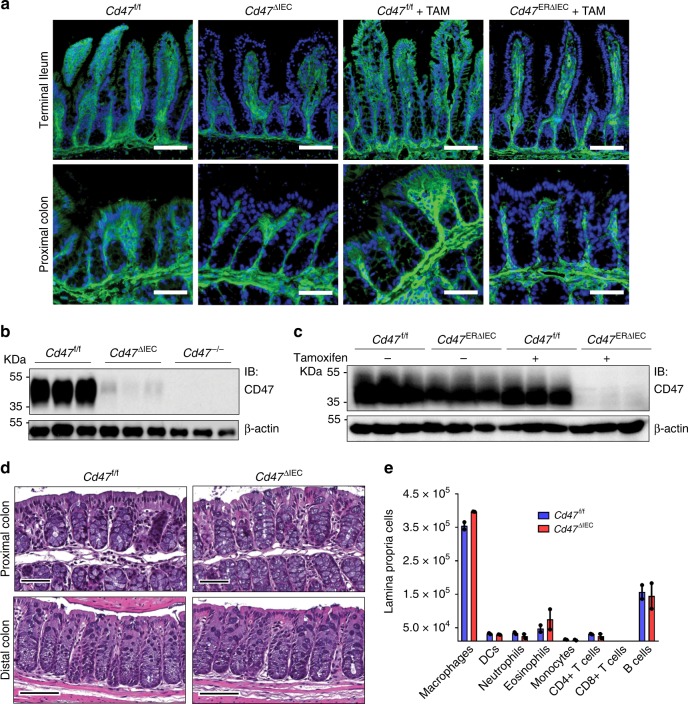
Loss of CD47 in IEC does not induce immune-mediated mucosal damage. **a**–**c** Naive *Cd47*^ΔIEC^ and tamoxifen-treated *Cd47*^ERΔIEC^ mice housed in pathogen-free conditions were analyzed for intestinal epithelial CD47 expression. *Cd47*^ERΔIEC^ mice were treated with tamoxifen to induce acute CD47 deletion in intestinal epithelial cells, and analyzed 2 weeks later. **a** Tissue sections from naive *Cd47*^ΔIEC^ and tamoxifen-treated *Cd47*^ERΔIEC^ mice were stained with anti-CD47 antibodies (green) with DAPI counterstain (blue). CD47 expression is absent in the epithelium but retained in the lamina propria and submucosa. Scale bars = 100 μm upper panels, 50 μm lower panels. **b** IECs  were isolated from the terminal ileum of naive mice. Protein lysates were analyzed by SDS–PAGE and immunoblot for CD47. **c** IECs were isolated from *Cd47*^ERΔIEC^ mice treated with vehicle (corn oil) or tamoxifen, and analyzed as in **b**. Results are representative of three independent experiments with 3–5 mice per group. **d** Paraffin-embedded colon tissue sections from *Cd47*^ΔIEC^ mice were stained with Hematoxylin and Eosin counterstain for histological examination. Gross mucosal architecture is intact in the absence of epithelial CD47 expression. Scale bars = 50 μm upper panels, 100 μm lower panels. **e** Colon tissue digests from naive *Cd47*^ΔIEC^ mice were stained and analyzed by flow cytometry, showing no significant differences in major lamina propria immune cell populations. Points represent individual samples each containing two mice (*n* = 4 mice per group). Data are means ± SEM and are representative of three independent experiments. Source data are provided as a Source Data file

As CD47 is ubiquitously expressed, understanding contributions of specific CD47-expressing cell types in mucosal wound-healing responses is critical to gain detailed mechanistic insights. Unfortunately, in vivo studies on CD47 function have been hindered by a lack of tissue-targeted, selectively deficient *Cd47* knockout mice. Given that mucosal wound healing is dependent on coordinated migration and proliferation of the intestinal epithelium, and CD47 is implicated in cell adhesion and migration in vitro^19^, we created mice with selective loss of CD47 in the intestinal epithelium by generating *Cd47* floxed mice (*Cd47*^f/f^), which were bred to mice constitutively expressing Cre under control of the *Villin* promoter (*Cd47*^ΔIEC^). Specific CD47 loss in IECs of *Cd47*^ΔIEC^ mice was confirmed by immunofluorescence labeling and western blotting (Fig. 2a, b).

As CD47 expression is described as an innate marker of self^4^, we anticipated that spontaneous immune-mediated destruction of the intestinal epithelium might develop in Cre-expressing mice. *Cd47*^ΔIEC^ mice weighed the same as littermate controls (Supplementary Fig. 1a) and exhibited no signs of spontaneous intestinal inflammation or epithelial barrier dysfunction. Furthermore, *Cd47*^ΔIEC^ mice did not show evidence of increased intestinal permeability to 4kD dextran (Supplementary Fig. 1b) consistent with normal barrier function. Histological examination of intestinal mucosa revealed normal architecture in *Cd47*^ΔIEC^ mice (Fig. 2d), and no significant inflammation or alterations in major tissue-resident immune cell populations assessed by flow cytometry (Fig. 2e). To verify that the lack of observed spontaneous inflammation in *Cd47*^ΔIEC^ mice is not owing to immune compensation during development^5^, *Cd47*^f/f^ mice were also bred to animals expressing an ERT2-Cre fusion protein under control of the *Villin* promoter^36^ to obtain mice with inducible loss of CD47 in the intestinal epithelium (*Cd47*^ERΔIEC^). Tamoxifen treatment of *Cd47*^ERΔIEC^ mice resulted in acute deletion of CD47 in IECs, which persisted 60 days post tamoxifen treatment (Fig. 2a, c). In agreement with results obtained from naive *Cd47*^ΔIEC^ mice, *Cd47*^ERΔIEC^ mice showed no signs of altered intestinal epithelial barrier function or pathologic mucosal inflammation, as evidenced by analysis of body weight, mucosal histology/immune cell populations, and barrier function (Supplementary Fig. 1c-e).

**Fig. 3:**
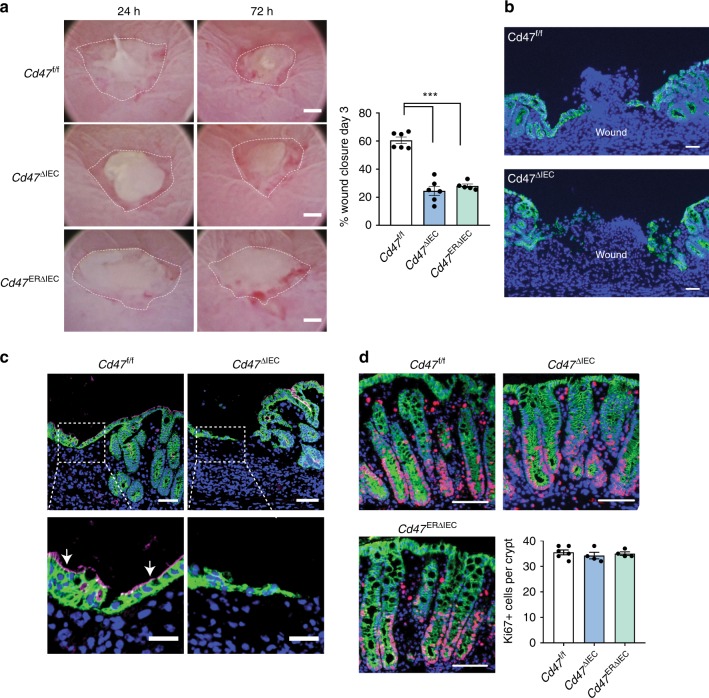
IEC-specific deletion of CD47 results in impaired mucosal healing. Utilizing a miniature video endoscope and biopsy scissors, 5–7 wounds were created in the dorsal aspect of the descending colon mucosa of anesthetized mice. *Cd47*^ERΔIEC^ mice were wounded 2 weeks after tamoxifen treatment. **a** Digital measurement of wound surface area at 24 and 72 h post wounding revealed a striking impairment in wound closure in *Cd47*^ΔIEC^ and tamoxifen-treated *Cd47*^ERΔIEC^ mice. Points represent mean value within all wounds from an individual mouse. Data are representative of two independent experiments with 5–6 mice per group. Data are means ± SEM. ***p < 0.001; one-way ANOVA. **b** Tissue sections taken from day 3 wounds were stained with the epithelial-specific marker E-Cadherin (green), plus DAPI counterstain (blue). Re-epithelialization of the wound is disorganized in the absence of CD47 (*Cd47*^ΔIEC^) compared with control *Cd47*^f/f^. **c** Tissue sections taken from day 3 wounds were stained with the epithelial-specific marker E-Cadherin (green), brush border protein Villin (magenta), and DAPI (blue). Insets of epithelial cells on top of the wound bed show polarized wound-associated epithelial cells (WAE) expressing Villin (arrows) in *Cd47*^f/f^ wounds while cells are not polarized in *Cd47*^ΔIEC^ wounds. Scale bars = 100 μm. **d** Ki67 staining of frozen sections of wounded colon mucosa 3 days post-wounding (red) revealed similar proliferation rates in crypt epithelial cells immediately adjacent to wounds in the absence of epithelial CD47. Sections were counterstained with E-Cadherin (green) and DAPI (blue). Scale bars = 50 μm. Points represent the average number of Ki67-positive cells for four crypts adjacent to wounds for each individual mouse. Data are means ± SEM and are representative of two independent experiments with 4–6 mice per group. Source data are provided as a Source Data file

Having established that IEC-targeted *Cd47-*deficient mice do not develop significant intestinal pathology under baseline conditions, we tested whether epithelial CD47 expression is required for mucosal wound healing. Consistent with our findings in *Cd47*^−/−^ mice and wild-type mice treated with anti-CD47 antibodies, *Cd47*^ΔIEC^ mice as well as tamoxifen-treated *Cd47*^ERΔIEC^ mice showed profound impairment in biopsy-induced wound closure between 1 and 3 days post wounding in comparison with littermate controls (Fig. 3a). Thin cross-sections of wound beds labeled with the epithelial-specific marker E-Cadherin and brush border protein Villin revealed the absence of polarized wound-associated epithelial cells expressing Villin in *Cd47*^ΔIEC^ mice compared with control *Cd47* ^f/f^ mice (Fig. 3b, c). In addition, examination of proliferation of cells in crypts adjacent to mucosal wounds isolated from *Cd47*^ΔIEC^ and tamoxifen-treated *Cd47*^ERΔIEC^ mice revealed similar numbers of Ki67-labeled epithelial cells (Fig. 3d), indicating similar levels of epithelial cell proliferation regardless of CD47 expression. Collectively, these findings indicate that epithelial CD47 promotes colonic mucosal wound healing by regulating IEC migration but not cell proliferation.

**Fig. 4:**
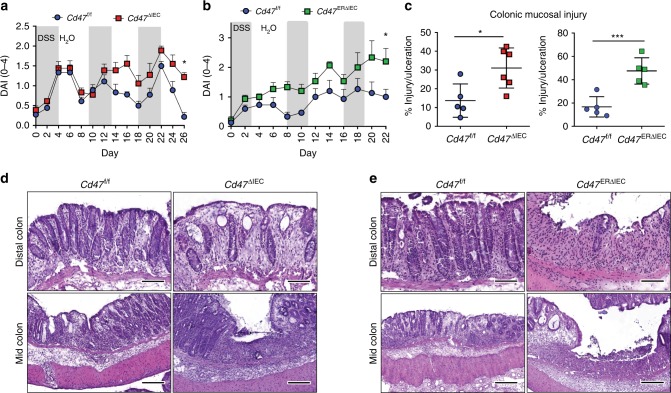
Loss of CD47 in IEC results in impaired recovery from DSS-induced colitis. Age- and sex-matched *Cd47*^ΔIEC^ and tamoxifen-treated *Cd47*^ERΔIEC^ mice were treated with three consecutive cycles of 2.5% DSS in drinking water for either 4 days (*Cd47*^ΔIEC^) or 3 days (*Cd47*^ERΔIEC^), followed by 5 days of water recovery. *Cd47*^ERΔIEC^ mice were treated with DSS 2 weeks after tamoxifen treatment. **a**–**b** Disease activity index scores are represented as an average of scores 0–4 for percent weight loss, stool consistency, and presence of blood in stools. Cyclical treatment of **a** *Cd47*^ΔIEC^ and **b** tamoxifen-treated *Cd47*^ERΔIEC^ mice with 2.5% DSS in drinking water, followed by a plain water recovery period, induced greater DAI scores in the absence of epithelial CD47. Data are representative of two independent experiments with 5–6 mice per group and are expressed as means ± SEM. **a** *p = 0.02, **b** *p = 0.036 by two-way ANOVA. **c** Histological scoring of hematoxylin and eosin (H&E)-stained tissue sections of colonic mucosa: percentage of injury/ulceration represents a ratio of the length of injured/ulcerated areas (≥ 50% crypt loss) relative to the entire colon length, as assessed in Swiss roll mounts of the entire colon. Results indicate greater damage in the absence of epithelial CD47. Points represent individual mice. Data are representative of two independent experiments with 5–6 mice per group and are expressed as means ± SEM. Significance determined by two-tailed Student’s *t* test. *p = 0.016, ***p = 0.001. **d**, **e** Representative H&E staining of colon tissue sections after three cycles of DSS/water revealed extensive crypt destruction in distal colon of *Cd47*^ΔIEC^ and *Cd47*^ERΔIEC^ mice. Large areas of ulcerated mucosa with granulocytic infiltrates were present in the mid colon in *Cd47*^ΔIEC^ and *Cd47*^ERΔIEC^ mice, in comparison with littermate controls. Scale bars = 100 μm upper panels, 300 μm lower panels. Results are representative of at least two independent experiments with 5–6 mice per group. Source data are provided as a Source Data file

In order to verify the observed, wound healing defects in *Cd47*^ΔIEC^ and tamoxifen-treated *Cd47*^ERΔIEC^ mice, we utilized a second model of chronic colonic mucosal injury and repair that employs cyclical administration of DSS in drinking water followed by recovery with water alone. When subjected to alternating cycles of DSS administration and water-only recovery, both *Cd47*^ΔIEC^ and tamoxifen-treated *Cd47*^ERΔIEC^ mice developed escalating clinical colitis scores (Disease Activity Index (DAI)) as determined by weight loss, stool consistency, and presence of blood in stools, in comparison with littermate controls (Fig. 4a, b). Colitis/DAI  was more pronounced following induced deletion of CD47 in tamoxifen-treated *Cd47*^ERΔIEC^ mice (Fig. 4b). Histological analysis of the colonic mucosa after three cycles of DSS and water administration revealed significantly worse mucosal injury and less repair in *Cd47*^ΔIEC^ mice and tamoxifen-treated *Cd47*^ERΔIEC^ mice, with increased crypt loss and mucosal ulceration evident in the distal colon (Fig. 4c–e). PMN/granulocytes were visualized in the inflamed colonic mucosa of both genotypes with prominent accumulations of PMN observed in *Cd47*^ΔIEC^ mice (Supplementary Fig. 2). These findings indicate that loss of CD47 results in impaired mucosal recovery associated with persistent inflammation in *Cd47*^ΔIEC^ mice in response to chronic DSS. In contrast, acute DSS treatment without recovery period did not produce statistically significant disease differences between groups (Supplementary Fig. 3a, b), further supporting impaired mucosal healing responses in *Cd47*^ΔIEC^ and *Cd47*^ERΔIEC^ mice.

### CD47 regulates focal adhesion-dependent IEC migration

**Fig. 5:**
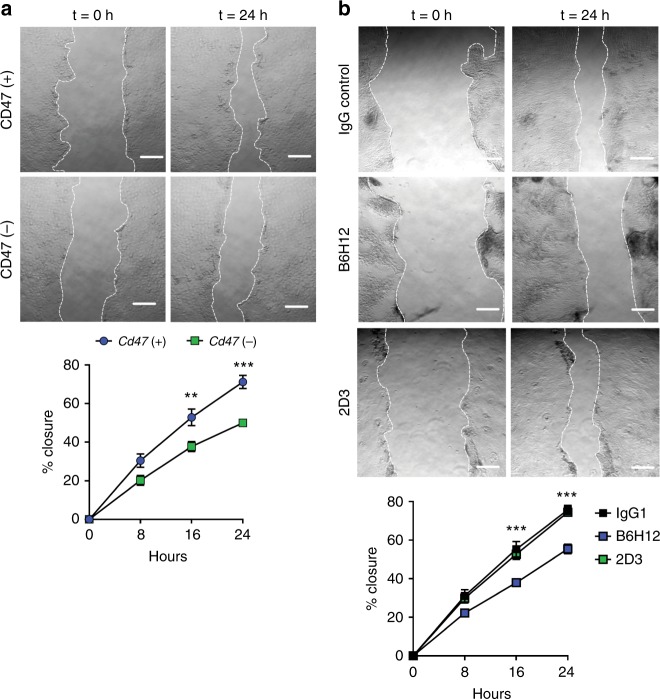
CD47 is required for wound repair in cultures of primary epithelial monolayers. **a** Primary epithelial cell monolayers derived from CD47-expressing (CD47( + )) or CD47-deficient (CD47(−)) murine enteroids were scratch-wounded and monitored for closure. CD47(−) epithelial monolayers showed significant impairment in reduction of scratch-wound surface area at 24 h post scratch. Edges of scratch wounds are indicated by dashed lines. Scale bars = 50 μm. **b** Primary epithelial cell monolayers derived from human stem cell-derived colonoids were scratch-wounded and treated with 10 µg/ml of either IgG control antibody, function-blocking anti-CD47 antibody (clone B6H12), or non-blocking anti-CD47 antibody (clone 2D3), resulting in the inhibition of cell migration upon blockade of CD47. **a**–**b** Results are representative of three independent experiments with three replicates per treatment group. Data are means ± SEM. Significance determined by two-way ANOVA, **p ≤ 0.01, ***p ≤ 0.001. Source data are provided as a Source Data file

To gain further insight into the molecular basis of delayed wound closure in epithelial cells lacking CD47, we analyzed wound closure in cultures of primary epithelial cells derived from either murine small intestinal enteroids or from human stem cell-derived epithelial colonoids. Murine CD47-deficient enteroid cultures were generated by treating *Cd47*^ERΔIEC^ intestinal enteroids with either vehicle (control) or 1 μm 4-hydroxytamoxifen (4-OHT) for 72 h to induce Cre-mediated recombination and stable CD47 knockdown (CD47( + ) or CD47(−), respectively). Deletion of CD47 in 4-OHT-treated enteroid cultures was verified by semiquantitative real-time PCR and western blotting (Supplementary Fig. 4a, b). Subsequently, 2D epithelial cell monolayers were generated from CD47( + ) or CD47(−) enteroids and subjected to scratch-wound healing assays. Although epithelial proliferation rates were similar between CD47( + ) and CD47(−) cultures (Supplementary Fig. 4c), time lapse imaging of scratch induced wounds revealed significantly delayed closure in monolayers lacking CD47 (Fig. 5a).

To further verify that blockade of CD47 impairs closure of wounds, we incubated human colonoid-derived epithelial monolayers with the well-characterized CD47-blocking antibody B6H12^37^. In comparison with IgG control, treatment with B6H12 significantly reduced wound closure, whereas incubation with the non-blocking anti-CD47 antibody 2D3 did not impede wound closure (Fig. 5b). Similar results were obtained by treating CD47-expressing murine epithelial monolayers with the blocking anti-CD47 antibodies miap301 or miap410 (Supplementary Fig. 5), further supporting a functional requirement for CD47 in epithelial scratch-wound closure. Collectively, these in vitro results support the above in vivo findings (Fig. 3) suggesting that IEC-expressed CD47 does not regulate cell proliferation but acts to promote epithelial cell migration in response to wound injury.

**Fig. 6:**
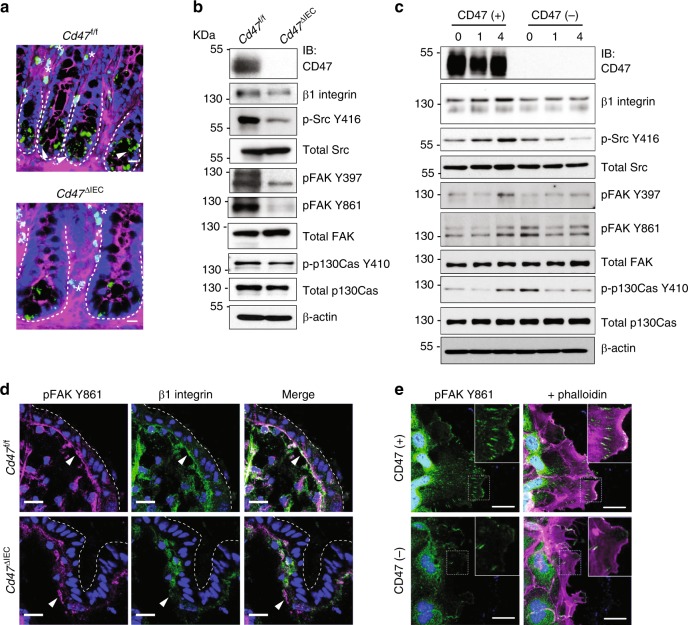
CD47 associates with β1 integrin and promotes focal adhesion formation. **a** In situ proximity ligation assay utilizing antibodies against CD47 and β1 integrin indicating close association between CD47 and β1 integrin in the colonic epithelium. Positive PLA signals are shown in green, beta-catenin in magenta and DAPI/nuclei in blue. Crypts are indicated by dashed lines. Arrowheads and asterisks (*) indicate positive PLA signals on IECs and immune cells in the lamina propria, respectively. Strong PLA signals are detected in IECs that are mainly concentrated at the base of the crypts in *Cd47*^f/f^ colon in contrast to very few PLA signals are observed in crypts IECs in *Cd47*^ΔIEC^ colons. Scale bars = 20 μm. The specificity of the PLA signals was evaluated in Supplementary figure 6 demonstrating no PLA signals in *Cd47*^−/−^ colons. **b** Whole-cell lysates from freshly isolated intestinal epithelial cells from *Cd47*^f/f^ and *Cd47*^ΔIEC^ mice were subjected to SDS–PAGE and immunoblot for signaling molecules downstream of β1 integrin-dependent cell adhesion, revealing decreased β1 integrin protein expression and reduced phosphorylation of Src^Y416^, FAK^Y397/Y861^, and p130CAS^Y410^ in cells from *Cd47*^ΔIEC^ mice. Results are representative of three independent experiments. **c** Murine enteroid-derived primary epithelial cell monolayers were scratch-wounded and harvested at the indicated time points, then analyzed by SDS–PAGE and immunoblot. CD47-deficient epithelial cells show a reduction in phosphorylated Src^Y416^, FAK^Y397/Y861^, and p130CAS^Y410^ upon wounding, whereas maintaining reduced β1 integrin protein baseline expression. **d** Epithelial cells immediately adjacent to wounds from *Cd47*^f/f^ and *Cd47*^ΔIEC^ mice were imaged by confocal microscopy, showing disrupted basal co-staining for phosphorylated FAK^Y861^ and β1 integrin (apical surface indicated by dashed line). Arrows indicate reduced colocalization of phospho-FAK^Y861^ and β1 integrin in the absence of CD47 expression. Scale bars = 10 μm. Results are representative of three independent experiments with three mice per treatment group. **e** Lamellipodia of CD47(−) cells exhibited fewer phosphorylated FAK^Y861^-positive focal adhesions in comparison with CD47-expressing cells (insets). Scale bars = 10 μm. Results are representative of three independent experiments with two independently derived enteroid culture lines. Source data are provided as a Source Data file

Previous in vitro studies have suggested that CD47 has a role in regulation of integrin-dependent leukocyte adhesion through direct interaction with αVβ3 integrin in neutrophils and α4β1/αLβ2 integrins in T cells^23,38^. In epithelial cells, it is well-established that integrin alpha–beta heterodimers containing the β1 subunit play a major role in epithelial cell migration through formation of focal adhesion complexes^17,18^, yet an interaction between CD47 and β1 integrins has not been investigated in the intestinal epithelium. We utilized an in situ proximity ligation assay (PLA) to test whether CD47 closely interacts with β1 integrin in murine colon. In Fig. 6a positive PLA signals were observed as green spots in IEC and immune cells with far fewer PLA signals observed in colonic crypts from *Cd47*^ΔIEC^ mice (Fig. 6a). Importantly, in Supplementary Fig. 6, we demonstrated specificity of PLA staining in using *Cd47*^−/−^ and *Cd47*^*+/+*^ mice. As expected, no PLA signals were detected on IEC or immune cells in the colonic mucosa from *Cd47*^−/−^ mice.

Previous studies have revealed that cell migration is dependent on temporal β1 integrin-dependent focal adhesion complex formation in epithelial cells with recruitment and autophosophorylation of FAK at nascent adhesions, followed by the Src kinase-dependent phosphorylation of the adaptor protein p130Cas^39–41^. We assessed whether this signaling pathway is altered in the absence of epithelial CD47 expression. Indeed, freshly isolated IECs from *Cd47*^ΔIEC^ mice revealed reduced β1 integrin protein expression in comparison with *Cd47*^f/f^ controls, reduced Src phosphorylation at residue Y416 and FAK tyrosine phosphorylation at residues Y397 and Y861 (pFAK^Y397^, pFAK^Y861^) as well as phosphorylation of p130Cas^Y410^ without change in total levels of Src and FAK (Fig. 6b). Similar results were obtained in wounded 2D epithelial cultures derived from murine enteroids, where CD47(−) monolayers had reduced β1 integrin protein, p-Src^Y416^, pFAK^Y397^, pFAK^Y861^, and p130Cas^Y410^ after wounding (Fig. 6c). Reduction of β1 integrin protein expression and altered pFAK^Y861^ in the absence of epithelial CD47 in vivo was confirmed through immunofluorescence staining and imaging of epithelium adjacent to healing wounds. Epithelial cells from *Cd47*^ΔIEC^ mice showed reduced basal staining for β1 integrin and pFAK^Y861^ and reduced colocalization of remaining β1 integrin and pFAK^Y861^ at the base of the epithelium (Fig. 6d). As β1 integrin-dependent signaling through FAK and p130Cas is necessary to direct formation and stabilization of adhesions in lamellipodia of migrating epithelial cells^16,40^, we analyzed focal adhesions in CD47(−) murine enteroid-derived epithelial cells. Immunofluorescence labeling of epithelial cells at the edges of healing wounds revealed typical focal adhesions in lamellipodia of leading-edge cells, visible as linear parallel pFAK^Y861^-staining structures connecting to the actin cytoskeleton in CD47( + ) cells. In contrast, very few pFAK^Y861^-staining focal adhesions were evident in lamellipodia of migrating CD47(−) (Fig. 6e). Similar results were obtained with pFAK^Y397^-staining lamellipodia of migrating CD47(−) cells that was irregular and punctate in comparison with CD47( + ) cells (Supplementary Fig. 7). In combination with observations of altered signaling downstream of β1 integrin in CD47-deficient cells, these results are consistent with a model of CD47-dependent regulation of β1 integrin function and integrin-dependent regulation of focal cell matrix adhesion proteins, which control epithelial cell motility and thus contribute to wound closure.

### CD47 regulates levels of TSP-1 and active TGF-β

**Fig. 7:**
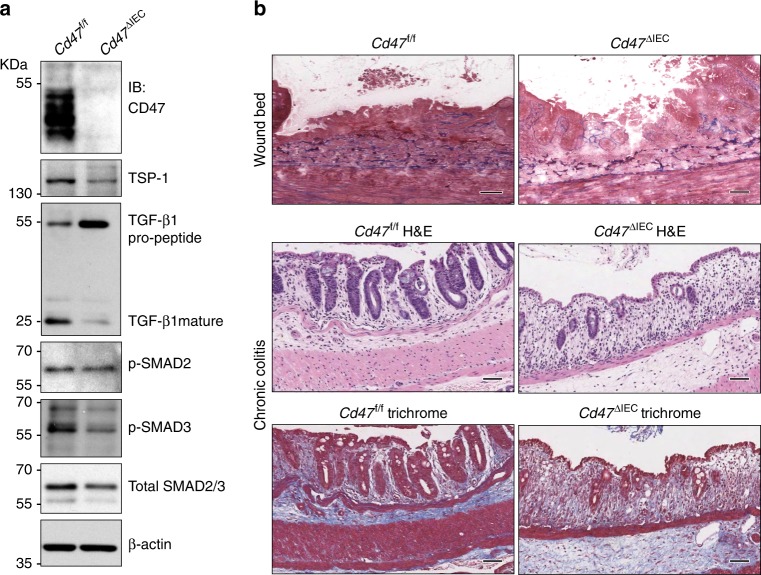
CD47 regulates thrombospondin-1, TGF-β1, and collagen deposition after injury. **a** Whole cell lysates from freshly isolated intestinal epithelial cells from *Cd47*^f/f^ and *Cd47*^ΔIEC^ mice were subjected to SDS–PAGE and immunoblot for thrombospondin-1/TSP-1, TGF-β1, and phosphorylated SMAD2 and SMAD3. Results are representative of three independent experiments. **b** Representative Masson’s trichrome staining of wounds beds and chronic DSS-colitis colons from *Cd47*^f/f^ and *Cd47*^ΔIEC^ mice. Scale bars = 50 μm. Results representative of three independent experiments with 5–7 mice per group. Source data are provided as a Source Data file

The CD47 soluble protein ligand TSP-1 has been reported to facilitate tissue repair, as *Tsp1*-null animals has been shown to have delayed dermal wound healing and enhanced susceptibility to DSS-colitis^30,42^. We thus investigated whether loss of CD47 had an impact on TSP-1 expression in IEC. As shown in Fig. 7a, reduced expression of TSP-1 was detected in IEC freshly isolated from *Cd47* ^ΔIEC^ mice in comparison with IEC from *Cd47*^f/f^ mice at baseline. As TSP-1 has been reported to be an activator of latent TGF-β1 to facilitate wound healing, re-epithelization and collagen synthesis^32,43^, we examined the effect of CD47 loss on activation of TGF-β1 and its downstream effectors as well as collagen deposition in colonic mucosa after injury. Freshly isolated IEC that lack CD47 expression had decreased expression of the mature cleaved form of TGF-β1 and accumulation of the immature pro-peptide (Fig. 7a). Total expression levels and phosphorylation of Smad2 and Smad3 were reduced similarly in IEC from *Cd47*^ΔIEC^ mice in comparison with *Cd47*^f/f^ controls (Fig. 7a). Consistent with these findings, we observed less collagen staining in *Cd47*^ΔIEC^ mice compared with *Cd47*^f/f^ controls after chronic DSS-induced colitis or biopsy-based wounds at day 3 (Fig. 7b). Collectively, these data strongly suggest that in addition to controlling focal adhesion activity, CD47 also promotes intestinal wound healing through effects on epithelial levels of TSP-1 and TGF-β1.

## Discussion

Despite the ubiquitous expression of CD47, very little is known about its function in non-hematopoietic cells. Previous studies employing models of inflammation in *Cd47*- null mice (*Cd47*^−/−^)^9–11,13,14^ or mice treated with CD47-neutralizing antibodies^34,35^ support both protective and pathological roles for CD47 expression, yet are highly model-dependent and limited in their ability to distinguish between the relative contributions of CD47-deficient cell types in vivo. Here, we provide in vivo mechanistic insights into the functions of CD47 on IEC during homeostasis and in response to chemically or mechanically induced wounds. We have established that CD47 expression is required for normal mucosal wound healing, as *Cd47*^−/−^ mice exhibited impaired colonic wound repair, an effect that was recapitulated in wild-type mice treated with CD47-neutralizing antibodies. It is possible that the observed wound healing defect in mice treated with anti-CD47 antibodies may, in part, be secondary to altered leukocyte responses, as CD47 has been reported to modulate leukocyte trans-endothelial migration^23,34^, cytokine production^44–46^, and bacterial killing^10^. Indeed, future studies utilizing cell type specific deletion of CD47 will be required to explore in depth the function of CD47 expression in other cell types during mucosal wound healing. However, here we show that epithelial CD47 expression is specifically required for mucosal wound healing, as *Cd47*^ΔIEC^ and tamoxifen-treated *Cd47*^ERΔIEC^ mice exhibited profound impairment of mucosal biopsy wound closure and develop escalating mucosal damage after cyclical DSS treatment and recovery. Similar to a previous report that used *Cd47*-null mice^47^, we observed no direct consequences of loss of CD47 on IEC in response to acute DSS treatment in *Cd47*^ΔIEC^ or *Cd47*^ERΔIEC^ mice in comparison with controls. The same study also reported that *Cd47*-null mice are protected against prolonged administration of DSS to model chronic colitis owing to attenuated granulopoiesis leading to reduced PMN accumulation in colonic mucosa. Herein, our findings indicate that loss of CD47 on IEC rendered mice more susceptible to DSS-induced chronic colitis. Although we used a different DSS protocol for chronic colitis that mimics relapsing–remitting course of inflammatory bowel disease^48,49^, a major difference with the previous study by Bian et al.^47^ is that we used animals with selective deletion of CD47 on IEC rather than total body knockouts. Given that all other tissues including immune cells express CD47, one would not expect to observe alterations in granulopoeisis as well as primary defects in the capacity of granulocytes to be recruited into the intestinal mucosa after biopsy or DSS treatment. Indeed, PMN/granulocytes were detected in the inflamed mucosa of all genotypes *Cd47*^ΔIEC^, *Cd47*^ERΔIEC^, and control *Cd47*^f/f^ mice after the last cycle of chronic DSS-induced colitis. We observed prominent infiltrates of PMN/granulocytes and large areas of crypt ulceration in *Cd47*^ΔIEC^ and *Cd47*^ERΔIEC^ mice, consistent with extensive epithelial damage and persistent inflammatory responses.

Studies employing transfer of CD47-deficient hematopoietic cells into wild-type hosts have informed the hypothesis that CD47 expression serves as an important marker of self, protecting CD47-expressing cells against errant destruction by phagocytic immune cells^3–5^. It was therefore unexpected to find a lack of immune-mediated mucosal damage upon acute deletion of intestinal epithelial CD47, yet this observation is consistent with initial studies reporting that *Cd47*^−/−^ mice are more or less healthy in pathogen-free conditions^10^. Although the reason for healthy *Cd47*^ΔIEC^ mice is not clear, certain macrophage functions could be dependent on licensing through exposure to CD47-expressing cells during development in order to sense CD47-deficient cells as non-self^5^. We found that both *Cd47*^ΔIEC^ and tamoxifen-treated *Cd47*^ERΔIEC^ mice did not experience spontaneous immune-mediated destruction of the intestinal epithelium. *Cd47*^ERΔIEC^ mice were more sensitive to DSS-induced colitis than ^ΔIEC^ mice, suggesting the possibility of a minor degree of innate immune compensation may occur during development. However, our observation that acute deletion of CD47 in the intestinal epithelium of adult *Cd47*^ERΔIEC^ mice is not sufficient to perturb intestinal homeostasis argues against this concept, as tissue-resident macrophages in these mice are continuously exposed to CD47-expressing epithelial cells prior to tamoxifen administration. Overall, our findings support a growing body of evidence suggesting that phagocytic activity directed at CD47-deficient cells likely involves other pro-phagocytic signals, or may be restricted to specific populations of macrophages and dendritic cells in vivo.

The process of mucosal barrier restitution upon wounding is dependent on coordinated migration of wound-adjacent epithelial cells^15^, whereas epithelial cell adhesion and migration is known to be dependent on the dynamic regulation of β1 integrin-containing focal adhesions^17,18^. Experiments conducted in vitro with 2D cultures of primary epithelial cells derived from murine enteroids or human colonoids confirmed that the absence or blockade of CD47 is sufficient to impair migratory capacity of wounded epithelial monolayers. The current studies provide mechanistic evidence that epithelial CD47 expression regulates mucosal wound closure in vivo by promoting signaling through a β1 integrin-dependent FAK-Src-p130Cas pathway^39,41,50^, thereby enabling focal adhesion complex formation and epithelial cell migration across wounded surfaces. Given that CD47 is expressed ubiquitously, these data suggest that CD47 may function as a fundamental regulator of integrin-dependent cellular processes in many tissues. In support of this, cellular processes as distinct as neuronal development^51^, smooth muscle cell chemotaxis^21^, and leukocyte adhesion and phagocytosis^23,37,38,52^ are reported to require both CD47 expression and the dynamic regulation of integrins. The exact nature of the interaction between CD47, β1 integrins and focal adhesions remains to be elucidated. In situ proximity ligation assays (PLA) confirmed close interaction between CD47 and integrin β1, as positive PLA signals were detected both in IEC and immune cells. These findings are consistent with other studies reporting direct interactions between CD47 and β1 integrins, α2β1 and α4β1^53,54^.

We detected reduced expression of β1 integrin protein and reduced/altered focal adhesions in CD47-deficient epithelial cells, suggesting that CD47 may facilitate cell migration by stabilizing β1 integrin protein expression. This would, in turn, promote the formation of, or prevent the dissolution of, β1 integrin-containing focal adhesion complexes^40,55,56^. Furthermore, we observed that in the absence of CD47 on IEC, phosphorylation of Src^Y416^ was reduced under basal conditions in freshly isolated IEC from *Cd47*^ΔIEC^ mice as well as in 2D primary IEC cultures in response to wound scratch injury. Our results in IEC corroborate previous studies in CD47-deficient neurons, demonstrating that autophosphorylation of Src was reduced, whereas overexpression of CD47 resulted in Src autophosphorylation^51^. Noteworthy, inhibition of Src has been reported to prevent CD47-promoted neuronal dendrite development as well as CD47-induced epithelial cell spreading, supporting a role for CD47 in epithelial cell migration and dendrite development via regulation of Src activity^51,57^.

The mechanism by which CD47 activates Src kinase is currently unknown. Given our findings of reduced phosphorylation of FAK in the absence of CD47 on IEC and reports of FAK acting as a molecular scaffold protein that recruits and activates Src, we speculate that FAK may play a key role in the regulation of Src by CD47. Of note, it has also been reported that Src-induced tyrosine phosphorylation of FAK is required for focal adhesion activity^50,58^. Collectively, these observations provide evidence for involvement of a functional axis consisting of CD47-FAK-Src in regulating CD47-dependent IEC migration. Interestingly, we found that the widely used anti-CD47 antibody B6H12, that inhibits binding of both ligands SIRP-α and TSP-1 to CD47, also resulted in reduced wound healing when applied to human stem cell-derived colonoids in vitro. Given that these human colonoids do not express SIRP-α (Supplementary Fig. 8), our results suggest that the effect of B6H12 mAb on epithelial wound healing may be owing in a large part to inhibition of the interactions between CD47 and TSP-1. However, we cannot exclude the possibility that binding of B6H12 mAb on CD47 may trigger intracellular signaling, as has been described in other types of cells (Supplementary Fig. 9)^59,60^.

Previous studies have reported that *Tsp1*-deficient mice displayed delayed wound healing and increased susceptibility to colitis^30,61^. Furthermore, TSP-1 is a major physiologic activator of latent TGF-β1, and TGF-β1 is described to be important for collagen deposition facilitating wound healing^29,32^. Here, we found that loss of CD47 on IEC resulted in reduced expression of TSP-1, cleaved/ active form of TGF-β1 as well as reduced phosphorylation of TGF-β1 downstream effectors, Smad2, and Smad3 in freshly isolated IEC from *Cd47*^ΔIEC^ mice. We also observed less collagen staining in *Cd47*^ΔIEC^ mice compared with *Cd47*^f/f^ mice three days after biopsy and following chronic DSS. It is tempting to speculate that decreased levels of expression of TSP-1 might contribute to observed phenotype in CD47-deficient IEC. These findings appear to be at odds with a report of improved wound repair in a dermal injury model using *Cd47*-deficient mice showing increased expression of TSP-1, activation of TGF-β1, increased Smad2/3 phosphorylation and increased deposition of collagen^29^; however, the latter used animals lacking CD47 in all cells of the body in contrast to our study that used mice with selective depletion of CD47 in IEC. Despite this difference, it is entirely possible that CD47 function in dermal epithelium is different than in IEC. Furthermore, both of these studies clearly support important contributions of CD47, TSP-1, and TGF-β1 in regulating epithelial repair after injury while highlighting the existence of tissue-specificity in the contributions of CD47 wound healing.

Although the precise mechanism by which CD47, TSP-1, and TGF-β1 cooperate to promote intestinal wound healing is unclear, it is possible that reduced levels of TSP-1 may lead to less activation of TGF-β1. This hypothesis is consistent with a previous report of *Tsp1*-deficient mice that exhibited aberrant skin wound healing, and topical application of a TSP-1-derived peptide (KRFK) was shown to enhance local expression of TGF-β1 in the wounds of *Tsp1*-deficient mice and rescued the wound healing process^32^. Moreover, TSP-1 and TGF-β1 have been reported to regulate FAK activity^62^ and ligation of CD47 by TSP-1 or specific TSP-1 peptides has been shown to regulate αvβ3 integrin-mediated cell spreading^63^. It is likely that TSP-1 and TGF-β1 might also functionally link CD47 to FAK. Collectively, these findings suggest the existence of a complex molecular network that regulates the CD47-dependent activation of FAK as well as IEC migration. Indeed, other CD47-associated proteins may also contribute to the process such as ubiquitin-related protein, PLIC-1 which has been suggested to regulate the actin cytoskeleton and cell migration^64,65^. The individual contributions of these signaling networks in regulating IEC migration after injury require further investigation.

Moreover, our observation that blockade of CD47 by antibody administration is sufficient to impair mucosal wound healing raises important considerations for therapeutic targeting of CD47. As immunotherapeutics directed against tumor-expressed CD47 are currently being tested in clinical trials, impaired wound healing may be an unintended consequence in cancer patients undergoing surgical resection, particularly in the gastrointestinal tract.

## Materials and methods

### Mice

*Cd47* knockout mice on a C57Bl/6 background were purchased from Jackson Laboratories and bred in-house at the University of Michigan in Ann Arbor. *Cd47* ^f/f^ mice were generated on a C57Bl/6 background from *Cd47*^tm1a(KOMP)Mbp^ knockout first ES cells obtained from the Knockout Mouse Repository at UC Davis (I.D. CSD44832) at the Mouse Transgenic and Gene Targeting Core at Emory University in Atlanta, GA. *Villin*^Cre^; *Cd47* ^f/f^ (*Cd47*^ΔIEC^), *Villin*^ERT2-Cre^;*Cd47*^f/f^ (*Cd47*^ERΔIEC^) and respective control littermates *Cd47*^f/f^ mice were bred in-house at the University of Michigan in Ann Arbor. Eight-week-old *Cd47*^ERΔIEC^ and control *Cd47*^f/f^ were injected intraperitoneally (i.p.) with 1 mg/100 μl of tamoxifen (T5648, Sigma) dissolved in sterile Corn oil (C8267, Sigma) for 5 consecutive days. Animals were used on days 15 after the last tamoxifen injection. For antibody neutralization experiments, 8-week-old male C57Bl/6 J mice were purchased from Jackson Laboratories. Mice were housed under specific pathogen-free conditions and used at 8–12 weeks of age. All experiments were approved and conducted in accordance with guidelines set by the University of Michigan Institutional Animal Care and Use Committee.

### Dextran sulfate sodium treatment

Mice were provided with 2.5% w/v DSS (40– kDa, 14489, Affymetrix) in drinking water ad libitum for indicated times followed by 5 days with normal drinking water^48,49^. Clinical disease assessment was obtained daily, with scores of 0–4 assigned for weight loss, stool consistency, and presence of blood in stools. The individual scores were added and the average recorded as the disease activity index (DAI). Higher values of DAI reflect increasing severity of colitis. At the end of the experiment, mice were sacrificed and colons harvested. Hematoxylin-Eosin (H&E) staining of sections of Swiss roll mounts of the entire colon (8-μm thick) was performed to quantify colonic mucosal injury. Percentage of injury/ulceration was calculated as a ratio of the length of injured/ulcerated area (≥ 50% crypt loss) relative to the entire colon length.

### Reagents

Function-blocking anti-CD47 antibody (clone B6H12.2) was purified from supernatants of hybridoma cells obtained from the American Tissue Culture Collection (ATCC)^52^. Antibodies against human SIRPαD1 (clones SAF10.1 and SAF17.2) or SIRPαD3 (SAF4.2) were produced in our laboratory^70^.

The following antibodies were purchased for Western Blot (WB), Immunohistochemistry (IF/IHC), Flow cytometry (FC) or neutralization assays: From AbCam: anti-integrin beta 1 (clone KMI6; ab95623; WB:1/1000), anti-Ki67 (ab15580; IF:1/250), Anti-thrombosponsdin-1 (ab85762; WB:1/500). From BD Pharmingen: anti-human b1 integrin (clone HUTS-21; Cat.556048 at 10 μg/ml), PerCP Anti-Mouse CD45 (clone 30-F11; Cat.557235; FC:1/200), PE-Cy7 Anti-Mouse CD3e (clone 145-2C11; Cat.552774; FC:1/200). BV421 Anti-Mouse Siglec F (clone E50-2440; Cat.562681; FC:1/200), BV510 Anti-Mouse CD4 (clone RM4-5; Cat.563106; FC:1/200). From Biolegend: Anti-mouse Ly6G (clone 1A8; Cat.127602; IHC:1/50). PE/Cy7 anti-mouse Ly6C (clone HK1.4; Cat.128018; FC:1/200), PerCP anti-mouse Ly6G (clone 1A8; Cat.127654; FC:1/200). From BioXCell: Purified rat IgG2A (clone 2A3; BP0089), miap301 (BE0270), and miap410 (BE0283) at 10 µg/ml. From Cell Signaling Technology: Anti-phospho-FAK (Tyr397; Cat.3283; WB:1/1000), Anti-FAK (Cat.3285; WB:1/1000), anti-phospho-p130CAS (Tyr410; Cat.4011; WB:1/1000), Anti-p130CAS (E1L9H; Cat.13846; WB:1/1000), phospho-Src (Tyr416) (clone D49G4; Cat.6943; WB:1/1000), Anti-Src (Clone 32G6; Cat.2123; WB:1/1000), phospho-SMAD2 (Ser465/Ser467 clone E8F3R; Cat.18338; WB:1/250), phospho-Smad3 (Ser423/425 Clone C25A9; Cat.9520; WB:1/500), Anti-Smad2/3 (Cat.5678; WB:1/1000). From GeneTex: Anti-Villin (GTX109940; IF:1/500). From Invitrogen: Alexa Fluor Secondary Antibodies for immunofluorescence (d:1/500), PE-Cyanine7 Anti-mouse F4/80 (clone BM8; Cat.25-4801-82; FC:1/200), PE Anti-mouse CD19 (clone 1D3; Cat.12-0193-83; FC:1/200), PE Anti-mouse CD11b (clone M1/70; Cat.RM2804; FC:1/200), APC Anti-mouse CD11c (clone N418; Cat.17-0114-82; FC:1/200), PE Anti-CD11b (clone M1/70; Cat.12-0112-83; FC:1/200), FITC Anti-CD8a (clone 53-6.7; Cat.11-0081-85; FC:1/200). From Millipore: Anti-phospho-FAK (Tyr 861; Cat.07-832; WB:1/1000). From Novus Biologicals: Anti-TGF-b1 (clone 7F6; NBP2-22114; d:1/250). From R&D systems: Anti-mouse CD47 (AF1866; WB:1/2000, IF:1/100), Anti-mouse E-Cadherin (AF748; IF:1/100), Rat anti- mouse Integrin beta 1 (MAB2405; IF:1/100). From Sigma Aldrich: Anti-beta actin (clone AC-15; A5441; WB:1/5000), Anti-GAPDH (G9545; WB:1/5000). From Thermofisher Scientific: Zenon-Alexa Fluor 488 Mouse IgG2a Labeling Kit (Z25102), Alexa Fluor 488-conjugated goat-anti-mouse (A11029; IF/FC:1/500), Anti-phospho-FAK (Tyr397; Cat.44-624 G; IF:1/100), Anti-phospho-FAK (Tyr 861; Cat.44-626 G; IF_d:1/100). Anti-mouse CD47 (clone 2D3; Cat.14-0478-82;10 μg/ml). Secondary antibodies for Western blot analysis were obtained from Jackson ImmunoResearch Laboratories.

### Intestinal enteroid and monolayer culture

Murine small intestinal epithelial enteroids/colonoids were created and maintained in culture. Mouse intestine was dissected and flushed with phosphate-buffered saline (PBS) and transferred to chelation buffer (2 mm EDTA, PBS) for 30 min. The intestine was shaken to remove crypt cells and then incubated in PBS with 43 mm sucrose and 55 mm sorbitol and filtered through a 70-μm filter^67^. Isolated intestinal crypts from untreated *Cd47*^ERΔIEC^ mice were embedded in Matrigel (BD Biosciences) (30 µl/well) and maintained in LWRN conditioned complete media supplemented with 50 ng/ml recombinant human EGF (R&D Systems) and 100× antibiotics–antimycotic (Corning). In some experiments, enteroid cultures derived from *Cd47*^ERΔIEC^ mice, were treated for 72 h with 1 μm z-4-hydroxytamoxifen (#H7904, Sigma Aldrich) in complete media to acutely knockdowm CD47 followed by passage and maintenance in tamoxifen-free complete media.

LGR5+ stem cell-derived human colonoids were provided by Jason Spence (University of Michigan) and maintained in Matrigel (BD Biosciences) in GMGF media composed of Advanced DMEM/F12, 10% FBS, 2 mm GlutaMAX-1, 10 mm HEPES, and 100 U/ml Penicillin/streptomycin (all from Invitrogen)^68^.

To generate 2D epithelial intestinal monolayers from murine or human 3D enteroids/colonoids, a single-cell suspension was obtained by resuspension in 0.05% Trypsin/0.5 mm EDTA and vigorously pipetting up and down. Trypsin was inactivated by adding 1 ml advanced DMEM/F12- containing 10% FBS. Dissociated cells were passed through a 40 µm cell strainer, then cultured in collagen type IV-coated 48-well tissue culture plates until confluency was achieved (~ 48 h). Murine 2D cultures were maintained in LWRN complete media supplemented with 50 ng/ml recombinant human EGF and antibiotics/antimycotic. Human 2D cultures were maintained in differentiation media containing GMGF media supplemented with 10 nm Gastrin and 1 mm *N-*Acetylcysteine (Sigma), 10 mm Nicotinamide, 10 µm SB202190 (StemCell Tech), 500 nm A83-01 (StemCell Tech), 10 ng/ml EGF (R&D systems)^68^.

### Wound healing assays

For in vitro experiments, 2D cultures of murine or human epithelial enteroids/colonoids were subjected to scratch wounding assays. Monolayers were cultured on collagen type IV (#C5533, Sigma)-coated 48-well tissue culture plates to confluency and scratched using a 10 μl pipette tip under suction. Medium was changed after wounding and video quantification of scratch-wound closure was performed by imaging wounds at 10 min intervals in an Axiovert Observer live cell microscopy system (Zeiss). Wound closure was quantified at the indicated time points using ImageJ software (National Institutes of Health), calculated as percent reduction of cell-free surface area compared with immediately after wounding (*t* = 0)^66^. For antibody-mediated neutralization experiments, antibodies were added immediately after wounding (time 0). Human primary epithelial monolayers were treated for 24 h with 10 μg/ml of IgG control (14-4714-82; Thermofisher), function-blocking anti-CD47 antibody (clone B6H12.2) or non-blocking anti-CD47 antibody (clone 2D3). Mouse primary epithelial monolayers were treated for 24 h with 10 μg/ml of rat IgG2A (clone 2A3), miap301 or miap410. For Western Blot analysis, multiple scratches were created to enrich the migratory fraction of cells before being collected in lysis buffer at indicated times.

For in vivo wounding experiments, a biopsy-based mucosal wound model was employed employed using a high-resolution video endoscope (Coloview Veterinary Endoscope, Karl Storz) equipped with biopsy forceps to create biopsy-induced injury of the colonic mucosa at five to seven sites along the dorsal aspect of the colon of anesthetized mice (i.p. injection of ketamine 100 mg/kg, xylazine 5 mg/kg). Wound healing was quantified at 24 h and 72 h after injury. Endoscopic procedures were viewed with high-resolution (1024 × 768 pixels) images on a flat-panel color monitor. Each wound region was digitally photographed at 24 h and 72 h, and wound areas calculated using ImageJ (National Institute of Health, USA). In each experiment, three to five wounds per mouse were quantified^66^. For antibody-mediated neutralization experiments, rat IgG2A (clone 2A3), miap301 or miap410 antibodies were intraperitoneally-administrated (400 µg in sterile PBS) or were injected directly into wound beds via veterinary endoscope 24 h post-wounding (10 µg in sterile PBS).

For immunofluorescence analysis, wounds were harvested from colons by punch biopsy, embedded and flash-frozen in OCT embedding compound, followed by sectioning (6 μm thick) and staining as indicated.

### Immunofluorescence

Frozen tissue sections (6μm thick) were fixed at room temperature in 4% paraformaldehyde, followed by blocking and permeabilization with 3% bovine serum albumin/0.5% Triton X-100 in PBS. Primary antibodies were incubated overnight at 4 °C in blocking buffer, followed by fluorescent secondary antibodies at room temperature for 1 h. Alexa Fluor 555 Phalloidin (A34055, Invitrogen) was used to stain F-actin and DAPI (4’,6-Diamidino-2-Phenylindole, Dihydrochloride) (D1306, Invitrogen) for nuclei. Slides were washed and mounted with Pro-Long Antifade Reagent (Thermo Fisher) prior to analysis as indicated.

In situ proximity ligation assay (PLA) was used to identify interactions between CD47 and integrin β1. Positive PLA signals, detected as a fluorescent dot by immunofluorescence microscopy, are produced when two labeled proteins are closely apposed within 40 nm. In situ PLA was performed on frozen tissue sections, fixed at room temperature in 4% paraformaldehyde, followed by blocking and permeabilization with 3% bovine serum albumin/0.5% Triton X-100 in PBS. DuoLink PLA probes and reagents (Sigma Aldrich) were used following the manufacturer’s instructions. Anti-Goat plus/DUO92003 was used to label anti-CD47 antibody and probemaker minus/DUO92010 for anti-integrin β1 antibody. Tissues were then stained with anti-beta-catenin antibody and DAPI. Positive and negative control experiments were performed using colonic mucosa from *Cd47*^+/+^ and *Cd47*^−/−^ mice, respectively.

### Western blot

Cells were lysed in RIPA buffer (150 mm NaCl, 1% NP-40, 0.5% deoxycholic acid, 0.1% SDS, 50 mm Tris pH 8.0), followed by sonication and centrifugation. Protein concentrations were quantified using BioRad DC Protein Assay (BioRad). Equal quantities of protein samples were loaded into 8% polyacrylamide gels for SDS–PAGE and transferred to polyvinylidene fluoride membrane. Membranes were blocked with either 5% dry milk/TBS/Tween-20 or 3% BSA/TBS/Tween-20 for phosphorylated proteins, followed by overnight incubation with primary antibodies at 4 °C. Membranes were then incubated with appropriate HRP-conjugated secondary antibodies for 1 h at room temperature, followed by development using Clarity Western ECL Substrate and image capture by ChemiDoc imager (BioRad). Uncropped and unprocessed scans of western blots are provided in the source data file.

### Flow cytometry

Lamina propria digests were prepared from whole colonic tissue by washing with chelation buffer to remove epithelial cells (10 mm EDTA in PBS), followed by digestion in RPMI media with 200 μg/ml Liberase TM (Roche Applied Science, Indianapolis, IN) and 200 U/ml DNase I (Sigma Aldrich). Cells were suspended in flow buffer (2% FBS/1 mm EDTA in DPBS) and incubated with the following fluorescent conjugated anti-mouse antibodies for 30 min at 4 °C: CD45, F4/80, CD11b, CD11c, Ly6G, Ly6C, Siglec F, CD19, CD3ε, CD4, and CD8α.

For detection of SIRPα, human epithelial colonoids were harvested by trypsinization, and washed twice in PBS. As positive controls, human peripheral blood polymorphonuclear leukocytes were isolated from whole blood obtained from healthy human volunteers, with approval from University of Michigan institutional review boards on human subjects. Human peripheral venous blood was collected and neutrophils were isolated using density gradient centrifugation on Polymorphprep (Cosmo Bio Usa Inc AXS1114683) at 450 × *g* for 35 min at 20 °C. Contaminating erythrocytes were removed by hypotonic lysis. Neutrophils isolated with this method were 97% pure and > 95% viable^69^. Cells were then suspended in 200 μl of PBS containing 2% FBS and incubated with 10 μg/ml of murine monoclonal antibodies against human SIRPαD1 (clones SAF10.1 and SAF17.2) or SIRPαD3 (SAF4.2)^70^ or anti-human CD47 (clone B6H12) mAb for 45 min at 4 °C. After incubation, cells were washed twice in PBS containing 2% FBS, and incubated for 30 min at 4 °C with an Alexa Fluor 488-conjugated goat-anti-mouse Ab.

To evaluate integrin β1 activation after antibody binding to CD47, human epithelial colonoids were harvested by trypsinization, and washed twice in HEPES/NaCl buffer (20 mm HEPES, 150 mm NaCl, 2 mg/ml d-glucose, pH 7.4). β1 integrin activation was detected by using anti-β1 integrin epitope antibody HUTS-21 previously labeled with Zenon-Alexa Fluor 488 Mouse IgG2a Labeling Kit (Cat# Z25102, Invitrogen), following manufacturer instructions. Cells were incubated either with 20 μg/ml of B6H12 mAb or after addition of 1 mm Mn^2+^, as a positive to induce integrin activation, in presence of anti-β1 integrin epitope Ab HUTS-21 to detect activation of β1 integrin. After 30 min (37 °C), cells were fixed in 4% paraformaldehyde for 10 min at room temperature followed by analysis on a NovoCyte flow cytometer (ACEA Biosystems, San Diego, CA). Data files were analyzed using FlowJo software (Tree Star, Ashland, OR).

### Statistics

Statistical significance was measured by Student's *t* test, one-way or two-way ANOVA using Graphpad Prism software. Significance was set as *p* ≤ 0.05. Results are expressed as means ± standard error of mean.

### Reporting summary

Further information on research design is available in the Nature Research Reporting Summary linked to this article.

## Supplementary information

Supplementary Information

Reporting Summary

Source Data file

## Data Availability

The authors declare that all data supporting the findings of this study are available within the paper and its supplementary information files, or from the corresponding author on reasonable request.
