# Supplementary material for: Epithelial CD47 is critical for mucosal repair in the murine intestine in vivo
Source: Nat Commun. 2019 Nov 1;10:5004. doi: 10.1038/s41467-019-12968-y (PMC6825175; doi:10.1038/s41467-019-12968-y)
Supplement: Supplementary file 1 — Supplementary Information [file 41467_2019_12968_MOESM1_ESM.pdf]

Supplementary Information

Epithelial CD47 is critical for mucosal repair in the murine intestine in vivo.

Reed M, Luissint AC et al.

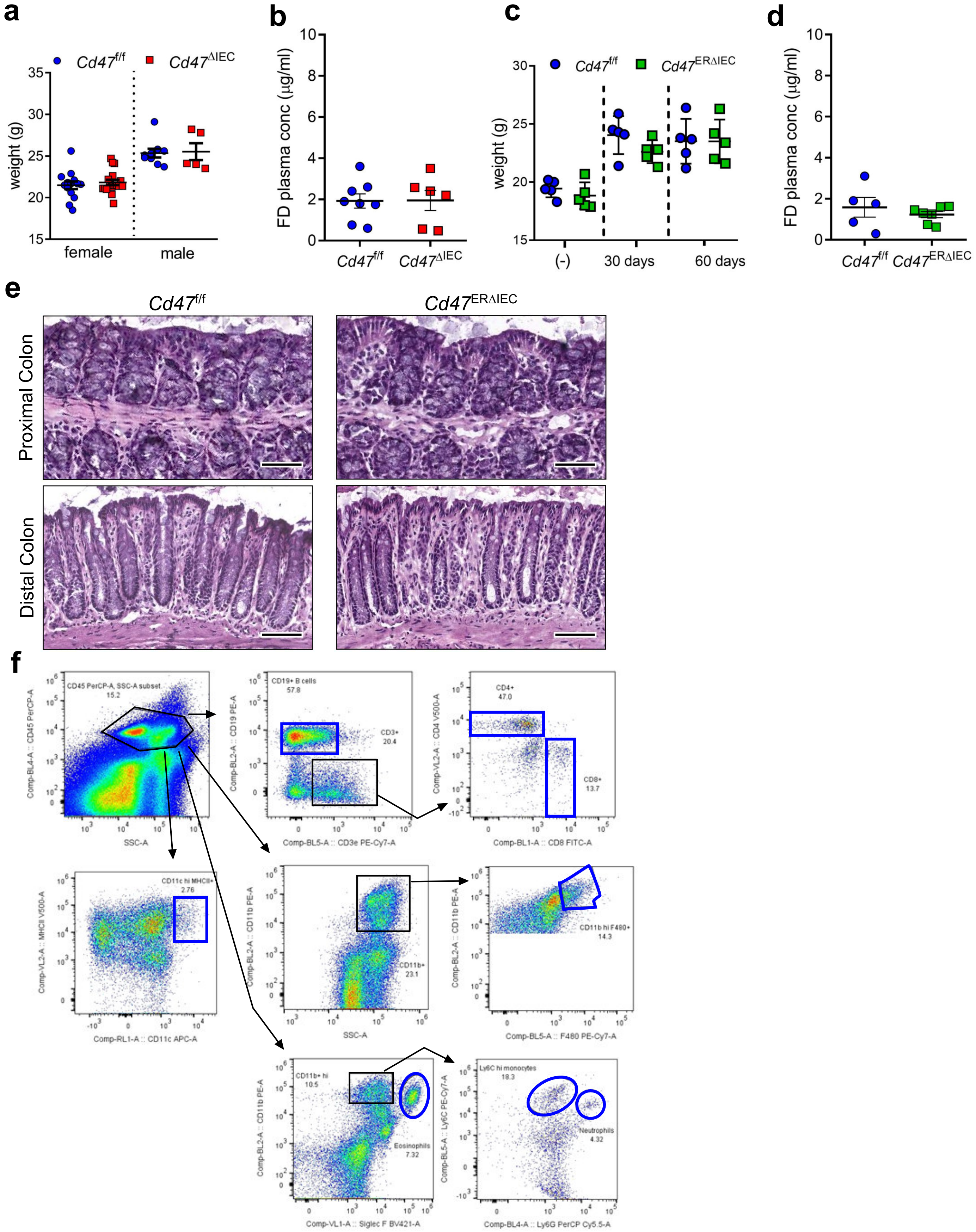

Supplementary Figure 1- Loss of CD47 in IEC does not alter barrier integrity or cause inflammation. **a**, *Cd47*$^{f/f}$ and *Cd47*$^{\Delta IEC}$ sex-matched littermates have similar body weight at 10 weeks of age in specific pathogen-free conditions. **b**, Intestinal permeability, measured by FITC-dextran gavage, is equivalent to controls in the absence of epithelial CD47. **c**, Body weight of *Cd47*$^{ER\Delta IEC}$ mice prior to tamoxifen treatment and at 30 or 60 days post-treatment does not differ from tamoxifen-treated controls, nor does intestinal permeability at 30 days post-treatment (**d**). Points represent individual mouse. Data are representative of 2 independent experiments and are expressed as means ± SEM. Differences are not significant. **e**, Gross mucosal architecture is intact in hematoxylin and eosin stained tissue sections of *Cd47*$^{ER\Delta IEC}$ mice 30 days after tamoxifen treatment. Scale bars = 50 µm upper panels, 100 µm lower panels. **f,** Flow Cytometry gating strategy for calculating numbers of resident leukocyte populations in lamina propria digests. Blue gates represent quantified populations. Black gates indicate parent gates, with black arrows pointing to child populations.

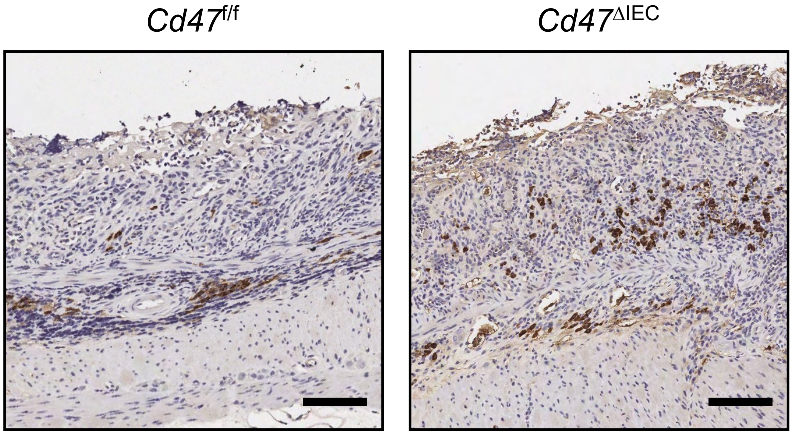

Supplementary Figure 2- PMN infiltration of colonic mucosa after DSS in *Cd47*$^{\Delta IEC}$ mice. Representative staining of PMN/granulocytes in colon tissue sections (ulcerated area) after three cycles of DSS/water. PMN were visualized by anti-Ly6G antibody (DAB (3, 3 -diaminobenzidine) HRP substrate, brown) and nuclei by hematoxylin (blue). Abundant PMN infiltrates were detected in the colonic mucosa of both *Cd47*$^{\Delta IEC}$ and *Cd47*$^{f/f}$ mice. Results are representative of 2 independent experiments with 3 mice per treatment group. Scale bars = 100 µm.

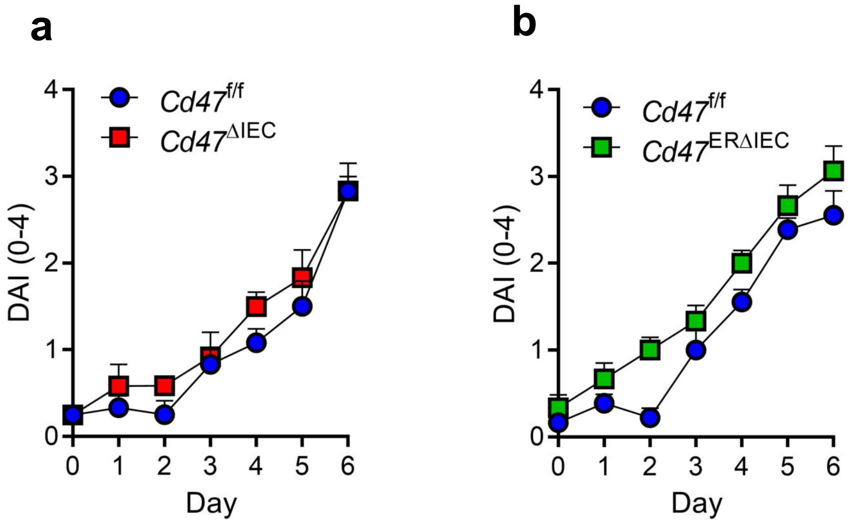

Supplementary Figure 3- IEC CD47 is not protective during acute DSS-induced colitis. Treatment of (**a**) *Cd47*$^{\Delta IEC}$ and (**b**) tamoxifen-treated *Cd47*$^{ER\Delta IEC}$ mice with 2.5% DSS in drinking water for 6 days with no recovery period produced similar disease activity index scores to respective controls. Data are means ± SEM. Results are representative of 2 independent experiments with 4 - 6 mice per treatment group and differences are not significant.

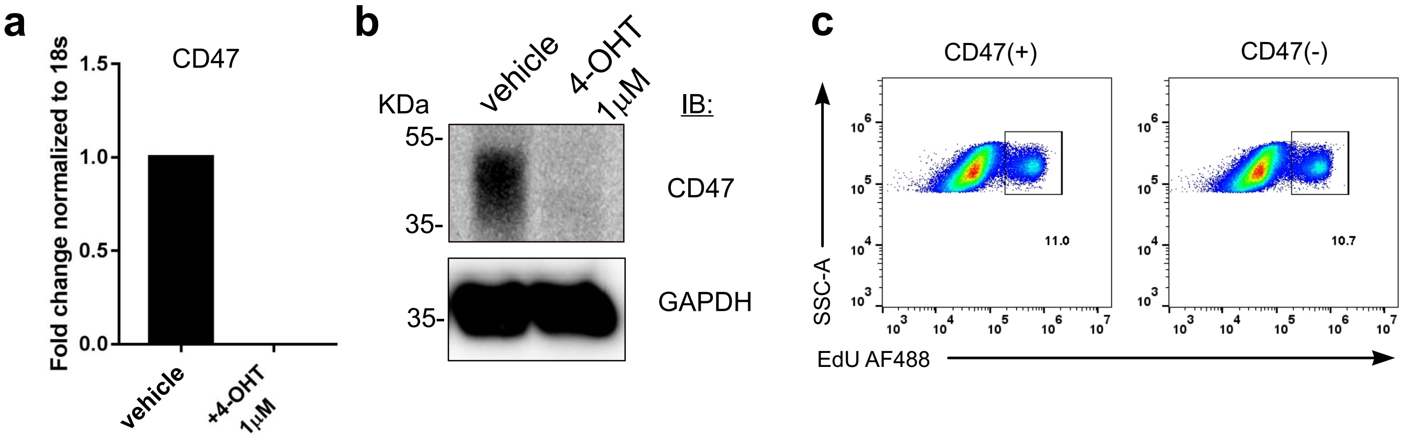

Supplementary Figure 4 - Deletion of CD47 in enteroid cultures from *Cd47*$^{ER\Delta IEC}$ mice. Murine epithelial enteroid cultures were generated from untreated *Cd47*$^{ER\Delta IEC}$ mice and treated in culture with vehicle or (z)-4-hydroxytamoxifen (4-OHT) as detailed in methods. Treated enteroids were passaged into tamoxifen-free culture and assessed for CD47 expression by semiquantitative qPCR (**a**) and immunoblot (**b**) 7 days later. **c**, Murine epithelial enteroids expressing CD47 (CD47(+)) or deficient in CD47 (CD47(-)) were pulsed with EdU (Click-iT™ EdU Cell Proliferation Kit, Invitrogen) for 30 minutes, then dispersed into single-cell suspension and stained for flow cytometry. The measured EdU uptake in CD47-deficient enteroids was equivalent to CD47(+) controls. Numbers represent the percent of total cells within gate. Results are representative of 3 experiments conducted with each of 2 independently-generated enteroid cultures.

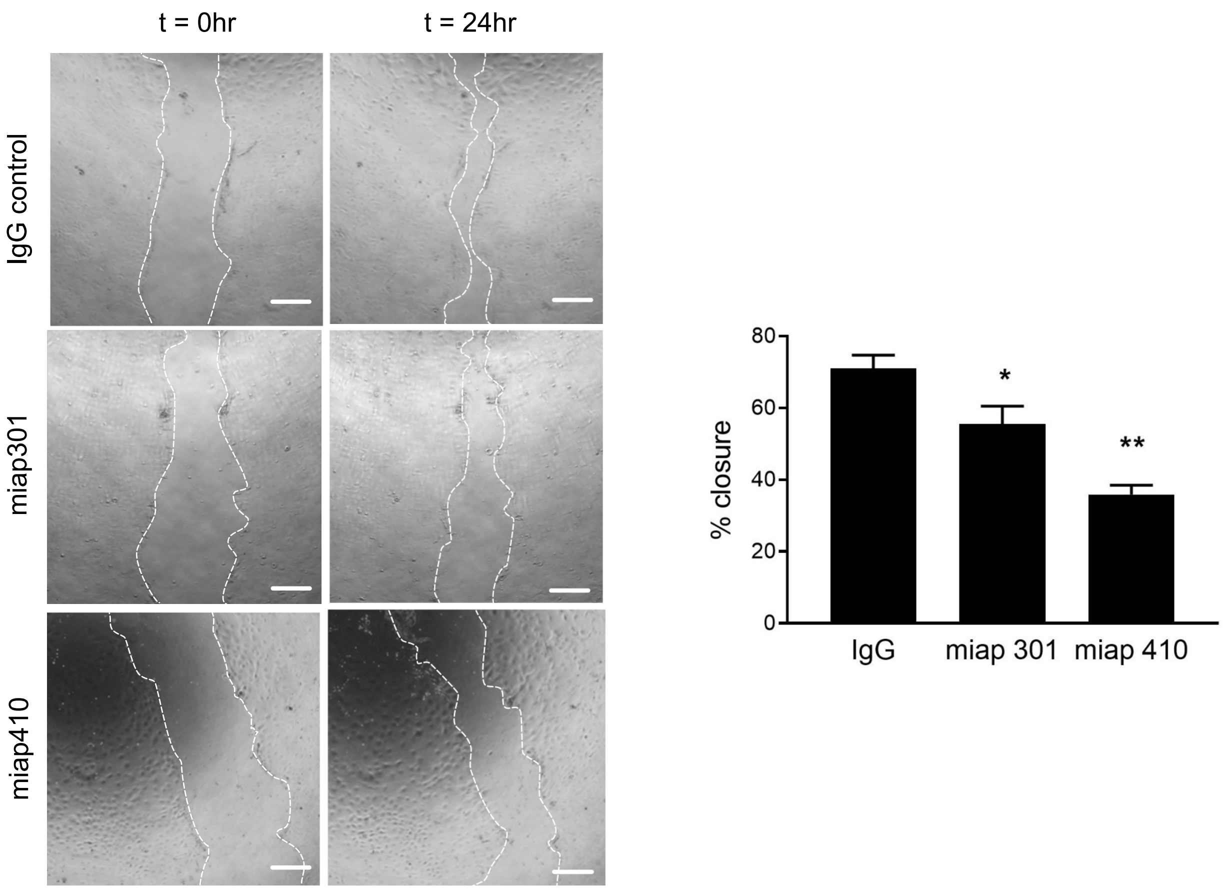

Supplementary Figure 5 – Anti-CD47 inhibits wound healing in primary mouse IEC monolayers. Intestinal epithelial enteroid cultures were generated from non-treated *Cd47*$^{ER\Delta IEC}$ mice as detailed in materials and methods. Confluent monolayers were scratched and immediately treated with 10 mg/ml rat IgG control antibody, anti-mouse CD47 clone miap301 or anti-mouse CD47 clone miap410. Epithelial monolayers treated with anti-CD47 antibodies showed significant impairment in reduction of scratch wound surface area at 24 hours post-scratch in comparison to isotype controls. Edges of scratch wounds are indicated by dashed lines. Scale bars = 50 μm. Results are representative of 3 independent experiments with at least 3 replicates per group. Data are means ± SEM. Significance determined by Two-tailed Student's t-test: *p=0.05, **p=0.002.

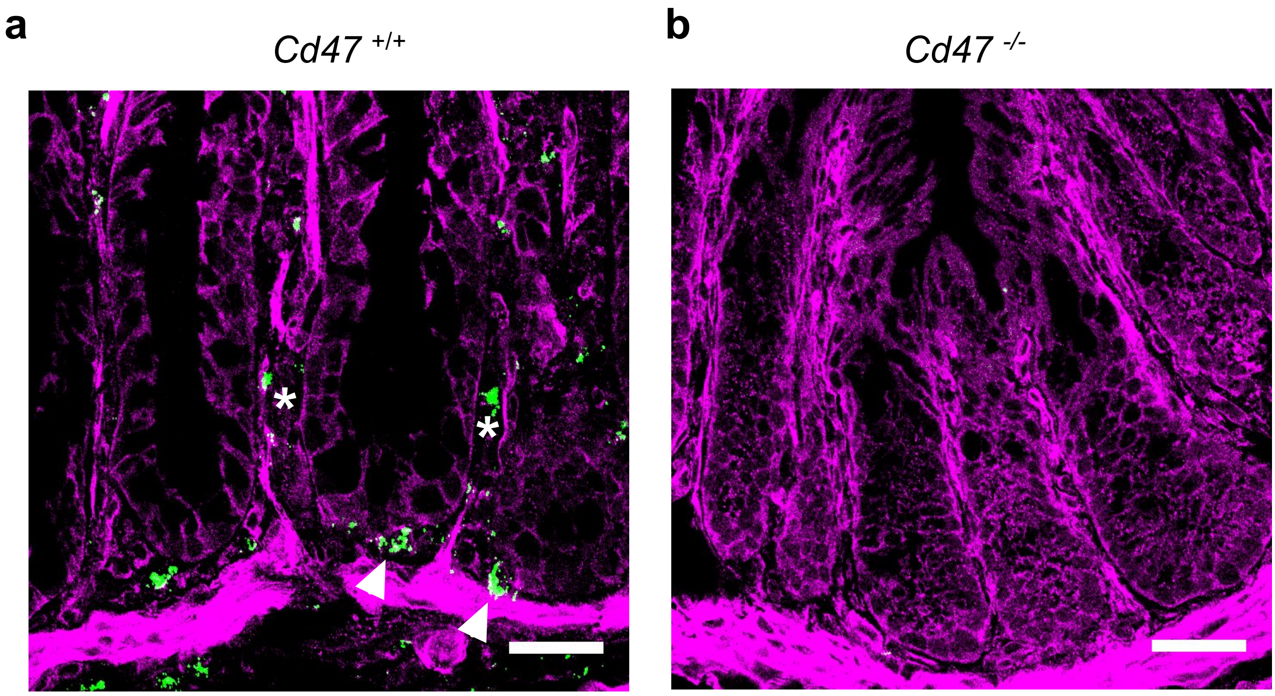

Supplementary Figure 6 - Close association between CD47 and β1 integrin in colonic mucosa. In situ proximity ligation assay (PLA) utilizing antibodies against CD47 and β1 integrin indicates interaction between CD47 and β1 integrin in the intestinal epithelium and lamina propria including immune cells. Arrowheads indicate positive PLA signal on IECs and asterisks (*) on immune cells in the lamina propria. (**a**) Positive PLA signals (green) are detected in WT colon (*Cd47*$^{+/+}$). (**b**) Absence of PLA signals in *Cd47*$^{-/-}$ colon. Anti-beta-catenin (magenta) was used as counterstain. Scale bars =100 µm. Results are representative of two independent experiments with 2 mice per group.

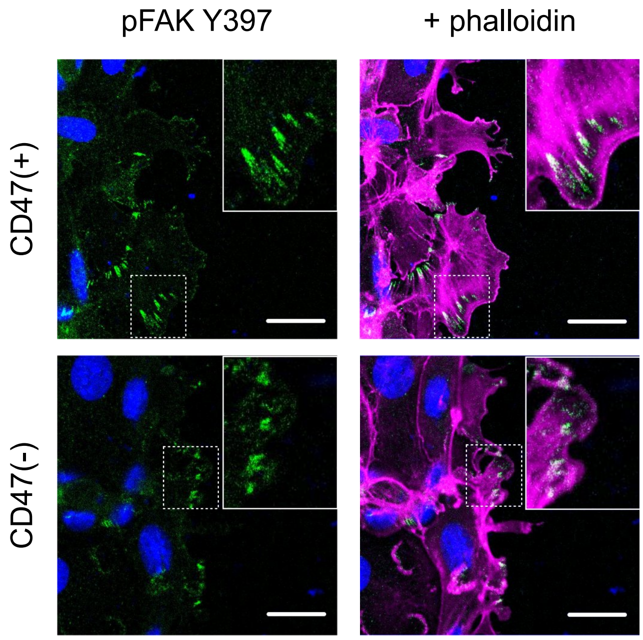

Supplementary Figure 7 - Epithelial CD47 promotes focal adhesion complex formation. Formation of focal adhesions by leading-edge cells from scratch-wounded murine enteroid-derived epithelial cell monolayers were analyzed by immunofluorescence staining and confocal microscopy. Linear FAK^{Y397}-positive structures visible in lamellipodia of migrating CD47(-) cells show altered morphology and reduced frequency in comparison to CD47-expressing cells/CD47(+) (insets). Scale bars =10 µm. Results are representative of 3 independent experiments with 2 independently-derived enteroid culture lines.

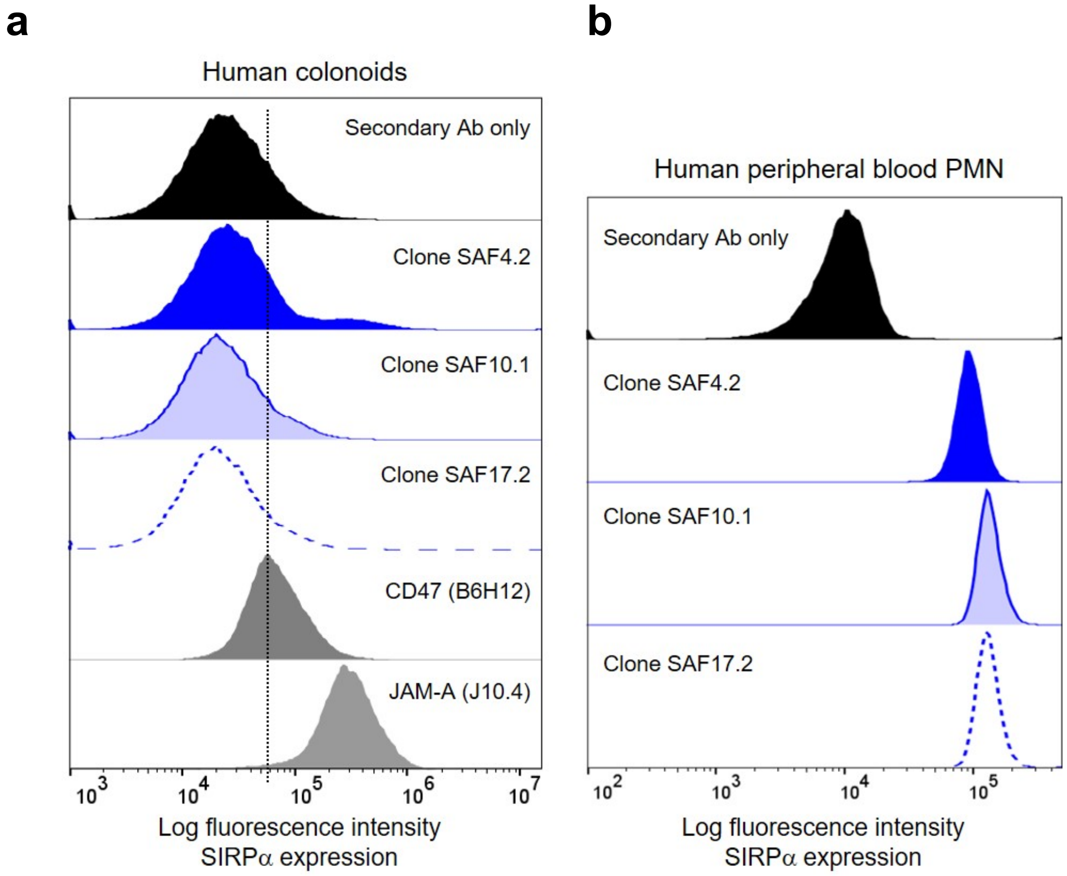

Supplementary Figure 8 - SIRPα is not expressed on primary human IEC. a, 2D epithelial monolayers of human colonoids were harvested by trypsinization. Cells suspensions were incubated with either 10 μg/ml of three distinct anti-human Signal-regulatory protein alpha/SIRPα mAbs (clones SAF4.2, 10.1, or 17.2), anti-human CD47 (clone B6H12) mAb or anti-human JAM-A (clone J10.4) mAb for 45 min at 4ºC. Samples were fixed and analyzed by flow cytometry. **b**, Human peripheral blood PMN were used as a positive control for the expression of SIRPα. Results are representative of two independent experiments conducted with independent human colonoid cultures and PMN isolation.

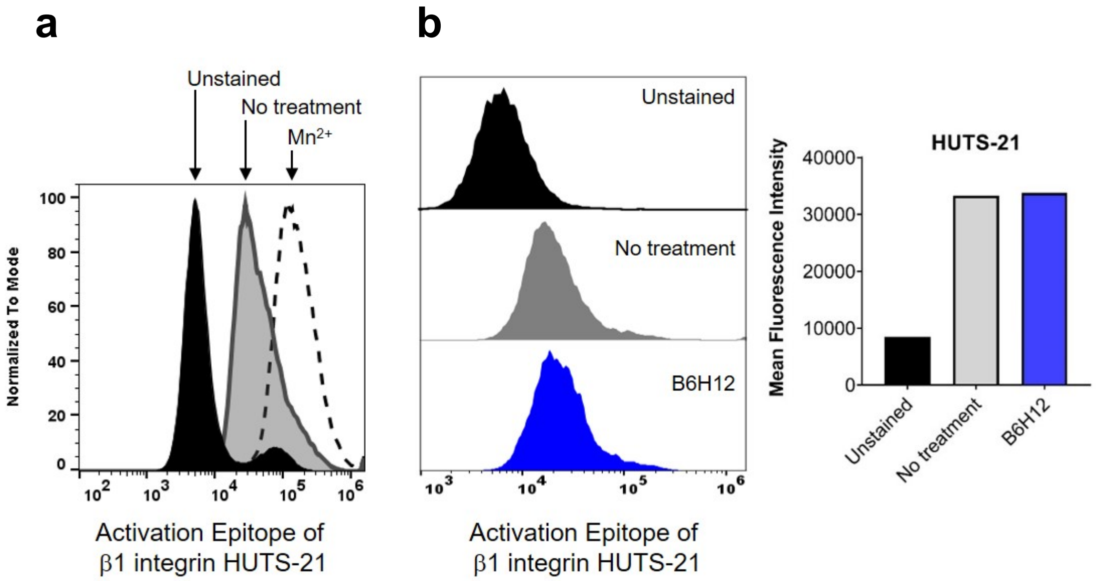

Supplementary Figure 9- Anti-CD47 mAb B6H12 does not activate β1 integrin in IEC. 2D epithelial monolayers of human colonoids were harvested by trypsinization. β1 integrin activation was monitored with the expression of the HUTS-21 epitope. Cells suspensions were incubated with 10 μg/ml of β1-Integrin activation epitope antibody HUTS-21 only (no treatment) or in presence of either (**a**) 1 mM Mn^{2+} (as a positive control) or (**b**) 20 μg/ml of anti-CD47 mAb B6H12 for 30 min at 37°C. Samples were fixed and analyzed by flow cytometry. Histograms display the mean fluorescence intensities. Unstained controls indicate the autofluorescence signal. Results are representative of two independent experiments conducted with independent human colonoid culture.
