# Supplementary material for: Epithelial CD47 is critical for mucosal repair in the murine intestine in vivo
Source: Nat Commun. 2019 Nov 1;10:5004. doi: 10.1038/s41467-019-12968-y (PMC6825175; doi:10.1038/s41467-019-12968-y)
Supplement: Supplementary file 2 — Reporting Summary [file 41467_2019_12968_MOESM2_ESM.pdf]

naturereserach

Corresponding author(s): Prof Charles A Parkos

Last updated by author(s): Sep 30, 2019

Reporting Summary

Nature Research wishes to improve the reproducibility of the work that we publish. This form provides structure for consistency and transparency in reporting. For further information on Nature Research policies, see Authors & Referees and the Editorial Policy Checklist.

Statistics

For all statistical analyses, confirm that the following items are present in the figure legend, table legend, main text, or Methods section.

n/a	Confirmed	
[ ]	[x]	The exact sample size (n) for each experimental group/condition, given as a discrete number and unit of measurement
[ ]	[x]	A statement on whether measurements were taken from distinct samples or whether the same sample was measured repeatedly
[ ]	[x]	The statistical test(s) used AND whether they are one- or two-sided *Only common tests should be described solely by name; describe more complex techniques in the Methods section.*
[ ]	[x]	A description of all covariates tested
[ ]	[x]	A description of any assumptions or corrections, such as tests of normality and adjustment for multiple comparisons
[ ]	[x]	A full description of the statistical parameters including central tendency (e.g. means) or other basic estimates (e.g. regression coefficient) AND variation (e.g. standard deviation) or associated estimates of uncertainty (e.g. confidence intervals)
[ ]	[x]	For null hypothesis testing, the test statistic (e.g. F, t, r) with confidence intervals, effect sizes, degrees of freedom and P value noted *Give P values as exact values whenever suitable.*
[x]	[ ]	For Bayesian analysis, information on the choice of priors and Markov chain Monte Carlo settings
[x]	[ ]	For hierarchical and complex designs, identification of the appropriate level for tests and full reporting of outcomes
[x]	[ ]	Estimates of effect sizes (e.g. Cohen's d, Pearson's r), indicating how they were calculated

Our web collection on statistics for biologists contains articles on many of the points above.

Software and code

Policy information about availability of computer code

Data collection	No software was used
Data analysis	Graphpad Prism software was used for statistical analysis<br>ImageJ software was used for Western blots and images analysis

For manuscripts utilizing custom algorithms or software that are central to the research but not yet described in published literature, software must be made available to editors/reviewers. We strongly encourage code deposition in a community repository (e.g. GitHub). See the Nature Research guidelines for submitting code & software for further information.

Data

Policy information about availability of data

All manuscripts must include a data availability statement. This statement should provide the following information, where applicable:
- Accession codes, unique identifiers, or web links for publicly available datasets
- A list of figures that have associated raw data
- A description of any restrictions on data availability

The authors declare that all relevant data supporting the findings of the study are available within the manuscript and its supporting information.

Field-specific reporting

Please select the one below that is the best fit for your research. If you are not sure, read the appropriate sections before making your selection.

[x] Life sciences [ ] Behavioural & social sciences [ ] Ecological, evolutionary & environmental sciences

For a reference copy of the document with all sections, see nature.com/documents/nr-reporting-summary-flat.pdf

Life sciences study design

All studies must disclose on these points even when the disclosure is negative.

Sample size	For experiments involving animals, a minimum of 5 animals per treatment group was used, as this is the number historically used to detect differences between experimental groups. For in vitro experiments, a minimum of 3 replicates was used to account for other sources of variation.
Data exclusions	No data were excluded from this study.
Replication	All experiments were performed independently at least two times (for large animal experiments) or three times (for in vitro experiments). All attempts at replication of the described findings were successful.
Randomization	Animals were allocated to experimental groups by assignment of their home cage to that group. This was done to enable the use of co-housed littermates, limiting other potential sources of variance (i.e. microbiome differences, environmental stress, etc.).
Blinding	All experimental animals were identified by ear tag number or assigned experimental identifier number. All data analysis was conducted by individuals blinded to the assigned experimental condition and genotype of the sample being analyzed.

Reporting for specific materials, systems and methods

We require information from authors about some types of materials, experimental systems and methods used in many studies. Here, indicate whether each material, system or method listed is relevant to your study. If you are not sure if a list item applies to your research, read the appropriate section before selecting a response.

Materials & experimental systems

n/a	Involved in the study	
[ ]	[x]	Antibodies
[x]	[ ]	Eukaryotic cell lines
[x]	[ ]	Palaeontology
[ ]	[x]	Animals and other organisms
[x]	[ ]	Human research participants
[x]	[ ]	Clinical data

Methods

n/a	Involved in the study	
[x]	[ ]	ChIP-seq
[ ]	[x]	Flow cytometry
[x]	[ ]	MRI-based neuroimaging

Antibodies

Antibodies used	Function-blocking anti-CD47 antibody (clone B6H12.2) was purified from supernatants of hybridoma cells obtained from the American Tissue Culture Collection (ATCC).<br>Antibodies against human SIRPαD1 (clones SAF10.1 and SAF17.2) or SIRPαD3 (SAF4.2) were produced in our laboratory.<br>The following antibodies were purchased for Western Blot (WB), Immunohistochemistry (IF/IHC), Flow cytometry (FC) or neutralization assays: From AbCam: anti-integrin beta 1 (clone KMI6; ab95623; WB:1/1000), anti-Ki67 (ab15580; IF:1/250), Anti-thrombospondin-1 (ab85762; WB:1/500).<br>From BD Pharmingen: anti human b1 integrin (clone HUTS21; Cat.556048 at 10ug/ml),PerCP Anti Mouse CD45 (clone 30-F11; Cat.557235; FC:1/200). PE-Cy7 Anti-Mouse CD3e (clone 145-2C11; Cat.552774; FC:1/200). BV421 Anti-Mouse Siglec-F (clone E50-2440; Cat.562681; FC:1/200). BV510 Anti-Mouse CD4 (clone RM4-5; Cat.563106; FC:1/200).<br>From Biolegend: Anti-mouse Ly6G (clone 1A8; Cat.127602; IHC:1/50). PE/Cy7 anti-mouse Ly-6C (clone HK1.4; Cat.128018; FC:1/200), PerCP anti-mouse Ly-6G (clone 1A8; Cat.127654; FC:1/200).<br>From BioXCell: Purified rat IgG2A (clone 2A3; BP0089), miap301 (BE0270), and miap410 (BE0283) at 10ug/ml.<br>From Cell Signaling Technology: Anti-phospho-FAK (Tyr397; Cat.3283; WB:1/1000), Anti-FAK (Cat.3285; WB:1/1000), anti-phospho-p130CAS (Tyr410; Cat.4011; WB:1/1000), Anti-p130CAS (E1L9H; Cat.13846; WB:1/1000), phospho-Src (Tyr416) (clone D49G4; Cat.6943; WB:1/1000), Anti-Src (Clone 32G6; Cat.2123; WB:1/1000), phospho-SMAD2 (Ser465/Ser467 clone E8F3R; Cat.18338; WB:1/250), phospho-Smad3 (Ser423/425 Clone C25A9; Cat.9520; WB:1/500), Anti-Smad2/3 (Cat.5678; WB:1/1000).<br>From GeneTex: Anti-Villin (GTX109940; IF:1/500).<br>From Invitrogen: Alexa Fluor Secondary Antibodies for immunofluorescence (d:1/500), PE-Cyanine7 Anti-mouse F4/80 (clone BM8; Cat.25-4801-82; FC:1/200). PE Anti-mouse CD19 (clone 1D3; Cat.12-0193-83; FC:1/200), PE Anti-mouse CD11a (clone M1/70; Cat.RM2804; FC:1/200), APC Anti-mouse CD11c (clone N418, Cat.17-0114-82; FC:1/200), PE Anti-CD11b (clone M1/70, Cat.12-0112-85; FC:1/200). FITC Anti-CD8a (clone 53-6.7; Cat.11-0081-85; FC:1/200).<br>From Millipore: Anti-phospho-FAK (Tyr 861; Cat.07-832; WB:1/1000). From Novus Biologicals: Anti-TGF-b1 (clone 7F6; NBP2-22114; d:1/250). From R&D systems: Anti-mouse CD47 (AF1866; WB:1/2000, IF:1/100). Anti-mouse E-Cadherin (AF748; IF:1/100), Rat anti-mouse Integrin beta 1 (MAB2405; IF:1/100).<br>From Sigma Aldrich: Anti-beta actin (clone AC-15; A5441; WB:1/5000), Anti-GAPDH (G9545; WB:1/5000).<br>From Thermofisher Scientific: Zenon-Alexa Fluor-488 Mouse IgG2a Labeling Kit (Z25102), Alexa Fluor 488-conjugated goat-anti-mouse (A11029; IF/FC:1/500), Anti-phospho-FAK (Tyr397; Cat.44-624G; IF:1/100), Anti-phospho-FAK (Tyr 861; Cat.44-626G; IF_d:1/100). Anti-mouse CD47 (clone 2D3; Cat.14-0479-82; 10ug/ml).

	Secondary antibodies for Western blot analysis were obtained from Jackson ImmunoResearch Laboratories.
Validation	All antibodies were used at concentrations and conditions recommended by the manufacturer. The information can be found in their official website. Customized antibodies against human SIRPα and CD47 (clone B6H12) were validated in previous studies. (Lee WY et al. J Immunol.2007.179:7741-50 and Liu Y et al, J Biol Chem.2001.276.40156-40166)

Animals and other organisms

Policy information about studies involving animals; ARRIVE guidelines recommended for reporting animal research

Laboratory animals	Mus musculus, strain background C57Bl/6. Both male and female littermates were used, between 8 and 12 weeks of age.
Wild animals	The study did not involve wild animals
Field-collected samples	The study did not involve samples collected from the field
Ethics oversight	All animal experiments were approved and conducted in accordance with guidelines set by the University of Michigan Institutional Animal Care and Use Committee.

Note that full information on the approval of the study protocol must also be provided in the manuscript.

Flow Cytometry

Plots

Confirm that:

[x] The axis labels state the marker and fluorochrome used (e.g. CD4-FITC).

[x] The axis scales are clearly visible. Include numbers along axes only for bottom left plot of group (a 'group' is an analysis of identical markers).

[x] All plots are contour plots with outliers or pseudocolor plots.

[x] A numerical value for number of cells or percentage (with statistics) is provided.

Methodology

Sample preparation	Mouse intestinal tissue was isolated and washed in chelation buffer to remove epithelial cells. Remaining tissue was minced and incubated in enzymatic digestion buffer, filtered, and stained in flow buffer on ice.
Instrument	NovoCyte flow cytometer model no. 3000, Acea Biosciences inc.
Software	Data was collected using NovoExpress software (Acea Biosciences), and analyzed using FlowJo v10.1.1
Cell population abundance	No cell sorting experiments were used in this manuscript.
Gating strategy	The gating strategy for lamina propria leukocyte subsets is shown in SupplementalryFigure 1f. Gating of positive populations was determined using unstained and single-stain controls.

[x] Tick this box to confirm that a figure exemplifying the gating strategy is provided in the Supplementary Information.
